# Distinct repeat architecture landscapes in the proteomes of protozoan parasites

**DOI:** 10.1093/nargab/lqag061

**Published:** 2026-06-27

**Authors:** Hirotaka Matsumoto, Jing Hong

**Affiliations:** School of Information and Data Sciences, Nagasaki University, Nagasaki, 852-8521, Japan; Omics AI Research Team, Advanced General Intelligence in Science Program (AGIS), TRIP Headquarters, RIKEN, Wako, Saitama, 351-0198, Japan; School of Tropical Medicine and Global Health, Nagasaki University, Nagasaki, 852-8523, Japan

## Abstract

Protozoan parasites cause major infectious diseases and pose persistent global health challenges, particularly the emergence of drug-resistant strains. Tandem repeats and other repetitive architectures are widespread in proteomes and have been implicated in host–parasite interactions, immune evasion, and antigenicity. However, repeat-containing proteins (RPs) exhibit highly diverse architectures that extend beyond simple motif reiteration, making their comprehensive and quantitative characterization challenging. In this study, we performed bioinformatics analysis of repeat architectures in protozoan proteins. In addition to the established repeat-detection approaches, we developed a new algorithm, Drepper, which quantifies repeat-architecture complexity. By integrating diverse repeat-related features, we clustered RPs across species and identified distinct groups associated with parasite lineages. Notably, we identified a high-complexity, repeat-rich (HCRR) cluster enriched in *Plasmodium* proteins and a low-complexity, repeat-rich (LCRR) cluster enriched in *Trypanosoma* and *Leishmania* proteins. Functional, evolutionary, and structural analyses revealed that LCRR cluster is enriched in flagellar-related proteins, low-complexity repeat architectures may be maintained through concerted evolution, and, compared with HCRR, it shows a greater tendency to adopt defined three-dimensional structures. Taken together, our results reveal lineage-specific strategies in protozoan repeat architectures and provide a quantitative framework for studying their biological and evolutionary roles.

## Introduction

Protozoan parasites are parasitic unicellular eukaryotes, including malarial parasites and *Leishmania*, many of which cause severe infectious diseases, including malaria caused by *Plasmodium* and leishmaniasis caused by *Leishmania*. These diseases result in a substantial number of deaths and are a major threat to global public health. Malaria alone affects hundreds of millions of individuals worldwide, with ~600 000 deaths annually [1]. In addition, an estimated 50 000–90 000 new cases of visceral leishmaniasis occur worldwide annually, with fatalities in over 95% of the cases when untreated [2]. In recent years, the emergence and spread of artemisinin-resistant malaria parasites have been reported, posing a new challenge for malaria control. The development of novel antiparasitic drugs and vaccines is urgently required to combat protozoan infectious diseases, including the expansion of drug-resistant strains. To achieve this, we require a detailed understanding of how protozoa invade host cells and evade or modulate host immune responses at the molecular and cellular levels.

Tandem repeats (TRs) are sequence architectures in which a specific amino acid subsequence, referred to as a repeat unit, is repeated iteratively. Proteins that contain TRs or other repetitive sequence architectures are collectively referred to as repeat-containing proteins (RPs). Early estimates suggested that ~14% of proteins, universally, are RPs [3], whereas more recent studies suggest that a substantially larger proportion of proteins contain repeat architectures [4]. Such repeat architectures form domains that mediate interactions with other proteins or nucleic acids [5]. Although repeat regions often lack stable tertiary structures and tend to be intrinsically disordered [6], they can function as scaffolds for molecular recognition and signal transduction [7]. Based on these properties, repeat architectures have been actively exploited in the design of artificial proteins [8].

Several RPs have been reported in protozoa, and many proteins in malaria parasites are composed of repetitive low-complexity sequences [9]. For instance, the circumsporozoite protein (CSP), a major target of malaria vaccines, contains prominent repeat regions. The central repeats of the CSP are required for sporozoite motility and exhibit spring-like mechanical properties [10]. Notably, the repeat sequences of CSP differ between *Plasmodium falciparum* and *Plasmodium vivax*, suggesting that a detailed understanding of repeat features is important for vaccine development [9]. Together with these findings, repeat-associated structural and functional features have been investigated in *Plasmodium* through computational analyses of its repetome [11]. In addition, repeat-containing proteins in protozoa are thought to play critical roles in processes such as cell adhesion, host cell invasion, and immune evasion [12]. Repeat features differ between intracellular parasites, extracellular parasites, and free-living protists, suggesting a link between repeat architectures and parasitic lifestyles. Moreover, TR-containing proteins are frequent targets of B-cell immune responses [13, 14], and high antigenicity of TR-containing proteins has been reported in *Leishmania* and *Trypanosoma* species [15, 16]. Although the numbers of RPs in *Leishmania* and *Trypanosoma* are lower than those in malaria parasites, these organisms often include RPs with exceptionally large TR regions. This observation highlights species-specific differences in repeat architectures and suggests that such differences may be linked to distinct parasitic strategies [16].

Repeat sequences are prone to rapid changes through expansion, contraction, and mutation and are therefore considered potential drivers of evolutionary innovation [17, 18]. In protozoa, increasing attention is being paid to the evolution of repeat sequences and their effects on parasitism, including species-specific differences in CSP repeats [9, 19–21]. In particular, repeat regions undergo concerted evolution, in which repeat units evolve cooperatively rather than independently. This mode of evolution may have a profound effect on host—parasite co-evolution [19].

Taken together, these findings indicate that RPs play an important role in protozoan parasitic strategies. However, the properties and functions of these RPs are highly diverse, and the biological significance of the repeat architectures in protozoa remains unclear.

Bioinformatics tools are essential for detecting and analyzing repeat architectures in protozoan amino acid sequences. To date, various algorithms have been developed to detect the TR regions, primarily based on sequence-processing approaches [22–25]. However, repeat architectures are often more complex than the simple reiterations of a single motif and may exhibit hierarchical or composite structures. Consequently, repeats cannot be treated as a uniform class and require multifaceted characterization and classification.

**Figure 1.**
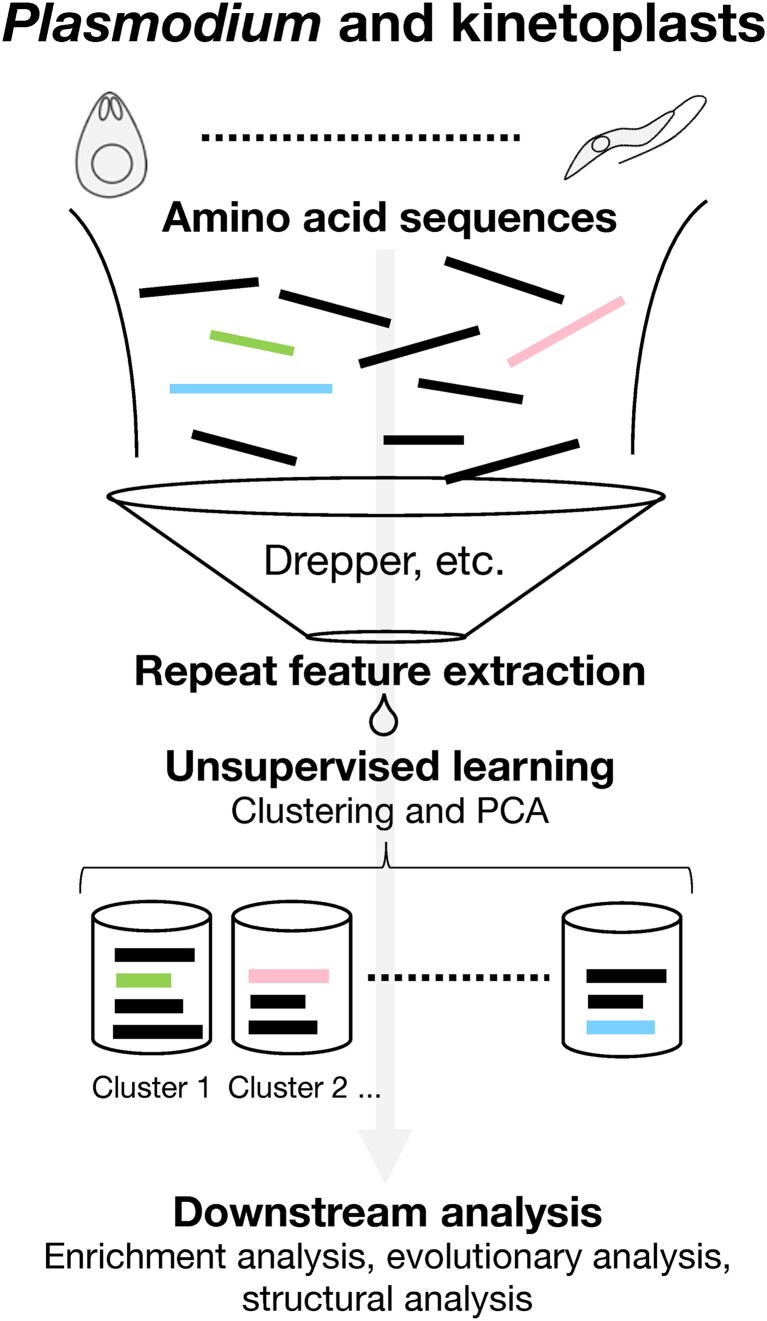
A graphical abstract illustrating the analytical workflow of this study.

In this study, we aimed to elucidate the diversity and characteristic features of repeats in protozoan proteins by quantitatively characterizing the repeat architectures from multiple perspectives and performing comprehensive analyses (Fig. 1). In addition to utilizing existing bioinformatics tools, we developed a novel algorithm, Drepper, based on self-dot plots, which enables quantitative assessment of repeat architecture complexity. Using a diverse set of repeat-related features, we clustered RPs and identified a high-complexity, repeat-rich (HCRR) cluster and a low-complexity, repeat-rich (LCRR) cluster; the former is enriched for proteins of *Plasmodium*, whereas the latter is enriched for proteins of *Trypanosoma* and *Leishmania*. This work represents the first comprehensive, quantitative comparison of repeat architecture complexity across protozoan lineages, uncovering lineage-specific repeat patterns that are difficult to capture using conventional repeat detection approaches.

## Materials and methods

### Overview

In this study, RPs were classified and subjected to downstream analyses based on quantitative features that characterize repeat architectures in amino acid sequences. To achieve this, it is essential to quantify repeat-related features from multiple perspectives. Accordingly, we developed a novel algorithm, Drepper, besides utilizing existing tools, to design and quantify diverse sets of repeat-related features. In the following sections, we first describe the features derived from the existing tool TANTAN, then introduce those computed using Drepper, and finally present the overall analytical workflow.

### Design of features based on TANTAN

Several sequence analysis tools have been developed to detect TR regions and their motif sequences in DNA and amino acid sequences [22]. In this study, we employed TANTAN, which can handle amino acid sequences [24]. TANTAN detects multiple TR regions within each amino acid sequence.

For a given amino acid sequence, suppose that $T$ TRs are detected. For the $i$-th TR ($i \in [1, T]$), TANTAN provides information on the start position $s_i$, end position $e_i$, repeat unit length $l_i$, repeat copy number $r_i$, motif sequence, and the actual sequences of individual repeat units. Based on the output of TANTAN, we designed the following TR-related features for each amino acid sequence:

**TRcount**: The number of detected TR regions corresponding to $T$.
**MaxTRS**: The maximum TR region size, defined as $\max _{i}(e_i - s_i)$. The index of the TR corresponding to MaxTRS is denoted by $i^{*} = \arg \max _i(e_i - s_i)$.
**RUP**: A metric that quantifies repeat unit purity within the MaxTRS region. This value is computed based on the average normalized edit distance as follows:
  \begin{eqnarray*}
1 - \frac{1}{\, _n C_2} \sum _{j,k} \left(\frac{d(x_j, x_k)}{l_{i^{*}}}\right),
\end{eqnarray*}
  where $n = r_{i^{*}}$, $x_j$ denotes the sequence of the $j$-th repeat unit of the $i^{*}$-th TR, and $d(a,b)$ represents the edit distance between sequences $a$ and $b$.
**MaxRUS**: The maximum repeat unit size, defined as $\max _i l_i$.

### Design of features based on Drepper

**Figure 2.**
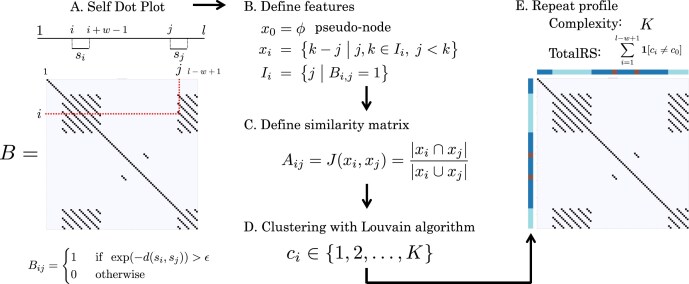
A schematic overview of the Drepper algorithm workflow.

We developed a novel algorithm, Drepper, based on self-dot plots, which enables quantitative characterization of repeat-structure complexity (Fig. 2). Below, we describe how the proposed algorithm quantifies the repeat-structure complexity and related properties for an amino acid sequence $s$ of length $l$. First, we defined the length-$w$ substring from position $i$ to $i+w-1$ as $s_i$ ($i\in [1,l-w+1]$) and constructed a $(l-w+1)\times (l-w+1)$ distance matrix $D$, where each entry $D_{i,j}$ is defined as the edit distance between substrings $s_i$ and $s_j$. We then defined a similarity matrix $S$ by $S_{i,j}=\exp (-D_{i,j})$. Next, we binarized $S$ to obtain a matrix $B$ such that $B_{i,j}=1$ if $S_{i,j}> \epsilon$, otherwise $B_{i,j}=0$ (Fig. 2A). In this study, we set $w=10$ and $\epsilon =0.2$.

Next, from the matrix $B$, we defined for each position $i\in [1,l-w+1]$, a feature vector $x_i$, as follows (Fig. 2B):


\begin{eqnarray*}
x_i \,\,=\,\, \bigl \lbrace k-j\bigm | j, k \in I_i,\,\,j < k\bigr \rbrace ,\,\,\,\,\,\,I_i=\bigl \lbrace j\bigm |B_{i,j}=1\bigr \rbrace .
\end{eqnarray*}

This represents the set of pairwise distances, $k-j$, between all ordered pairs of indices $(j, k)$ satisfying $B_{i,j} = 1$, $B_{i,k} = 1$, and $j < k$. If a given substring $s_i$ has a very large number of similar substrings (i.e. the number of indices $j$ satisfying $B_{i,j}=1$ is very large), computing all pairwise distances becomes computationally expensive. In such cases, we restrict the construction of $x_i$ to a subset of indices $j$ corresponding to the 50 smallest values of $|i-j|$.

We then added a pseudo-data point representing unique, non-repetitive subsequences by introducing $x_0=\phi$, where $\phi$ denotes an empty set. We defined a similarity matrix $A$ among the $(l-w+2)$ feature vectors using the Jaccard index (Fig. 2C):


\begin{eqnarray*}
A_{i,j}=J(x_i, x_j)= \frac{|x_i \cap x_j|}{|x_i \cup x_j|}.
\end{eqnarray*}

We defined $J(\phi ,\phi )=1$. Subsequently, we binarized the similarity matrix $A$ at a threshold of 0.5 to obtain $A^{\prime }$. Next, $A^{\prime }$ was treated as the adjacency matrix of a graph, and clustering was performed using the Louvain algorithm. This procedure yielded cluster assignments $c_i$ for each node $(i \in [0, l-w+1])$ (Fig. 2D). Here, $c_1,\ldots ,c_{l-w+1}$ represent the clustering results for each position in the self-dot plot, whereas $c_0$ corresponds to the cluster ID, representing regions that do not correspond to repeats.

As described above, positions within a sequence can be clustered based on information from the off-diagonal elements of the self-dot plot, enabling the quantitative characterization of properties such as the complexity of repeat structures in the amino acid sequence. Based on this framework, we designed the following features to quantify repeat-related features (Fig. 2E).

**Complexity**: The complexity of repeat regions within an amino acid sequence, quantified as the diversity of clusters, is defined as: $K = \max _i(c_i)$.
**TotalRS**: The total extent of repeat-associated regions within an amino acid sequence is computed as: $\sum _{i=1}^{l-w+1} \mathbf {1}\!\left[c_i \ne c_0\right]$.

These metrics were validated for stability using simulated datasets (details are provided in the Supplementary Text and Supplementary Fig. S2).

### Datasets and preprocessing

We analyzed the amino acid sequences from 12 parasitic species of malaria parasites and some kinetoplastids, focusing on strains with relatively well-curated genome annotations. The target parasitic species and their corresponding strains are listed in Table 1 (rows 1–12). Amino acid sequence data for malaria parasites were obtained from PlasmoDB [26] (release 68), and those for kinetoplastids were obtained from TriTrypDB [27] (release 68). Amino acid sequences encoded by short contigs, mitochondrial genomes, or apicoplast chromosomes were excluded from the analysis.

**Table 1.** Species, strain, and lifestyle information

Abbreviation	Species	Strain/Isolate	Lifestyle
Pfalciparum3D7	*Plasmodium falciparum*	3D7	Parasitic
PvivaxP01	*Plasmodium vivax*	P01	Parasitic
PknowlesiH	*Plasmodium knowlesi*	H	Parasitic
LmajorFriedlin	*Leishmania major*	Friedlin	Parasitic
LdonovaniHU3	*Leishmania donovani*	HU3	Parasitic
LmexicanaMHOMGT2001U1103	*Leishmania mexicana*	MHOM/GT/2001/U1103	Parasitic
LamazonensisPH8	*Leishmania amazonensis*	PH8	Parasitic
LmartiniquensisLEM2494	*Leishmania martiniquensis*	LEM2494	Parasitic
CfasciculataCfCl	*Crithidia fasciculata*	Cf-Cl	Parasitic
TbruceiTREU927	*Trypanosoma brucei*	TREU927	Parasitic
TcruziBrazilA4	*Trypanosoma cruzi*	Brazil A4	Parasitic
PconfusumCUL13	*Paratrypanosoma confusum*	CUL13	Putatively parasitic
Cvelia	*Chromera velia*	CCMP2878	Free-living
Bsaltans	*Bodo saltans*	CYKH01	Free-living
Ddiscoideum	*Dictyostelium discoideum*	AX4	Free-living
Scerevisiae	*Saccharomyces cerevisiae*	S288C	Free-living

*P. confusum* represents an early-branching kinetoplastid and may retain ancestral parasitic or host-associated features [28].

For each of the 12 species, the repeat-related features were computed for each amino acid sequence using both TANTAN and Drepper. Together with the sequence length (Length), seven features were calculated for each amino acid sequence. Features from all species were integrated to construct a data matrix $X$ of size $N \times 7$ for $N$ amino acid sequences. All features except RUP were log-transformed. In addition, amino acid sequences with TotalRS equal to zero were removed prior to downstream analyses. The final dataset comprised $N$ = 6380 sequences of RPs.

Using this feature matrix, amino acid sequences were clustered using the $k$-means algorithm, and principal component analysis (PCA) was performed on $X$. To enable fine-grained characterization of RP properties based on repeat-related features, we set the number of clusters to a relatively large value, $K = 7$. These clustering results were stable and robust (details are provided in the Supplementary Text and Supplementary Fig. S1).

In addition, protein sequences from four free-living species listed in Table 1 (rows 13–16) were included as comparative datasets. The protein sequences for these species were obtained from CryptoDB [29] (release 68) or from the corresponding GenBank assemblies (Supplementary Table S1). The same repeat-related features were then calculated for these additional datasets. Subsequently, the resulting feature vectors were projected onto the previously constructed PCA space, and each amino acid sequence was assigned to the closest cluster based on the cluster centroids derived from the initial clustering analysis.

**Figure 3.**
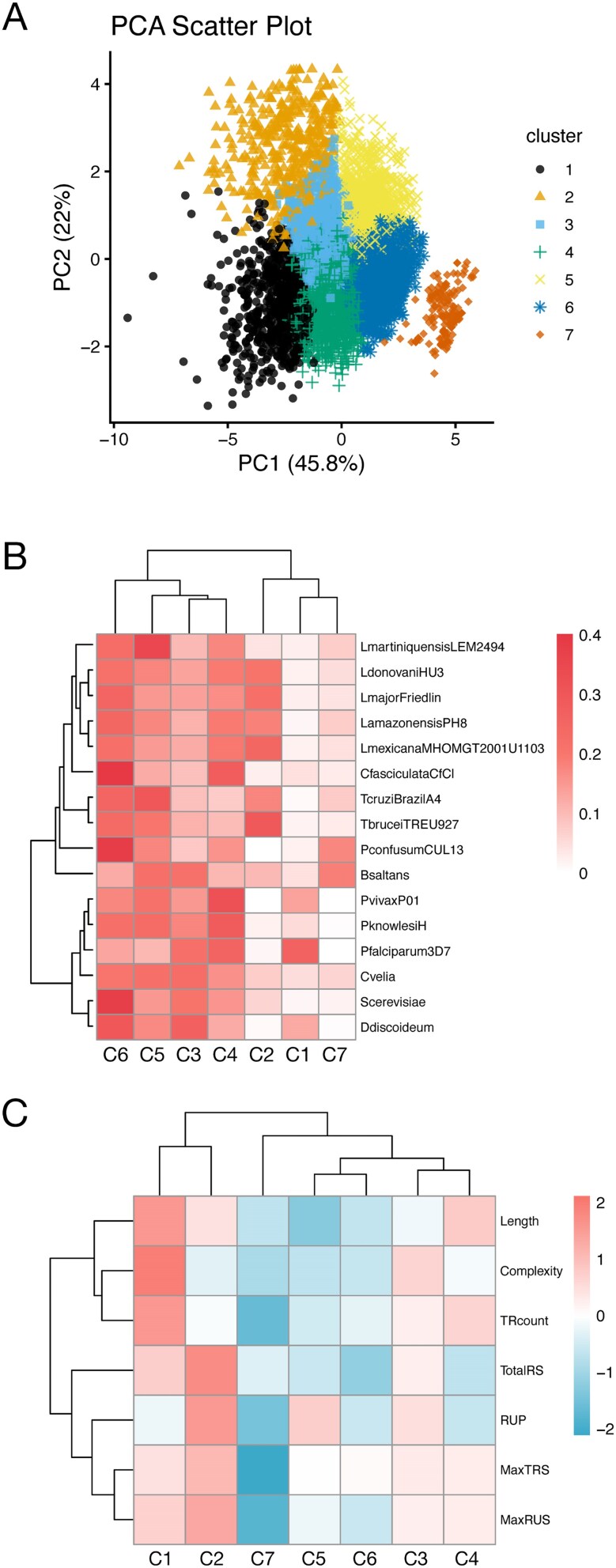
(**A**) Visualization of amino acid sequence clustering based on repeat-related features projected onto the PCA space. (**B**) Heatmap showing the species composition of each cluster, including 4 comparative species. The row dendrogram corresponds to the species tree inferred using OrthoFinder [30]. (**C**) Heatmap of standardized mean values of repeat-related features for each cluster.

The relationships among the 16 species used in this study were inferred by constructing a species tree using OrthoFinder [30] and are shown in Fig. 3B.

### Enrichment analyses

#### Species enrichment analysis

Let $C$ denote a $12 \times 7$ count matrix summarizing the number of RPs from each of the 12 species assigned to each cluster. We tested whether a specific group of parasitic species was enriched in a given cluster, using the following procedure.

Let $g$ be a vector representing species group membership, where $g_i = A$ if species $i$ belongs to the focal species group, otherwise $g_i = B$. Let $k$ denote the cluster of interest; define a vector $x$ such that $x_i = C_{i,k}$ represents the number of RPs from species $i$ assigned to the cluster $k$. The total number of RPs for species $i$ is defined as $n_i = \sum _{j=1}^{7} C_{i,j}$.

We tested whether species with $g_i = A$ exhibited a significantly higher proportion of RPs assigned to cluster $k$, i.e. $x_i / n_i$, compared to species with $g_i = B$. Statistical testing was performed using a generalized linear model based on a binomial distribution implemented in R (glm()). The response variable was specified as cbind$(x_i, n_i - x_i)$, the explanatory variable was $g_i$, and the model was fitted with family = binomial. The $p$-value was obtained from the significance of the regression coefficient associated with $g_i$.

#### Signal peptide prediction and analysis

Signal peptides were predicted for all amino acid sequences from 16 species, including outgroups, using SignalP 6.0 [31]. For each species, sequences classified as non-RPs and those assigned to each of the seven clusters were counted separately according to the presence or absence of signal peptides.

Using the non-RP set as a control, we assessed whether signal peptides were significantly enriched or depleted within each cluster by performing two-sided Fisher’s exact tests. For each test, both the $p$-values and the direction of change (enrichment or depletion) were recorded. The $p$-values were calculated independently for each species and subsequently adjusted for multiple testing using a Bonferroni correction.

#### GO enrichment analysis

GO annotations for each species were obtained from GAF files downloaded from PlasmoDB and TriTrypDB. We focused on 1963 GO terms that appeared at least once among the 6380 RPs analyzed.

For each of the 1963 GO terms, we counted the number of RPs associated with that term in each cluster. The enrichment of a given GO term within a specific cluster was assessed using a one-sided Fisher’s exact test. For each cluster, raw $p$-values were computed for all GO terms and subsequently adjusted for multiple testing using a Bonferroni correction.

Two control sets were used for comparison: (i) the set of amino acid sequences belonging to all clusters other than the target cluster and (ii) the set of all amino acid sequences not belonging to the target cluster, including non-RPs, that appeared at least once in the GAF files. The enrichment $p$-values were computed separately, using each control set.

#### Protein signature enrichment analysis

Protein signatures present in each amino acid sequence were predicted using InterProScan (release 5.76–106.0) [32]. We focused on 6442 signature accessions that appeared at least once among the 6380 RPs.

For each signature accession, we counted the number of RPs associated with that accession, in each cluster. When the same signature accession was predicted in multiple regions of a single amino acid sequence, it was counted only once for that sequence. The enrichment of a given signature accession within a specific cluster was assessed using a one-sided Fisher’s exact test, followed by a Bonferroni correction to obtain adjusted $p$-values.

Two control sets were used: (i) the set of amino acid sequences belonging to all clusters other than the target cluster and (ii) the set of all amino acid sequences not belonging to the target cluster, including non-RPs, for which at least one protein signature was predicted by InterProScan. The enrichment $p$-values were computed separately using each control set.

### Evolutionary analysis

Evolutionary analyses were restricted to nine kinetoplastid species. Orthologous relationships were inferred using OrthoFinder [30] and orthogroups that were frequently assigned to C2 were extracted. Among the amino acid sequences belonging to the same orthogroup, all repeat motifs predicted by TANTAN in the *Leishmania major* Friedlin strain were treated as candidate repeat motifs.

For each candidate motif, all amino acid sequences in the same orthogroup were used as references, and the motif sequence was used as a query in BLASTP searches to enumerate all sub-sequences matching the motif. An e-value threshold of $1\times 10^{-5}$ was applied, followed by the filtering of hits with bit scores below 100. The resulting sequences were output in FASTA format.

Similar sequences for each motif were listed, and those motifs for which similar sequences were detected in all four *Leishmania* species (LmajorFriedlin, LdonovaniHU3, LamazonensisPH8, and LmexicanaMHOMGT2001U1103) were selected; the corresponding hit sequences were subjected to comparative sequence and phylogenetic network analyses. Multiple sequence alignment was performed using MAFFT [33]. The resulting alignments were visualized globally using ggmsa [34] and sequence logos were generated for each species using ggseqlogo [35]. Identical sequences within the same species were collapsed into a single representative sequence, and sequence similarity was visualized as a phylogenetic network using SplitsTree [36].

### Structural analysis

Predicted structures for *P. falciparum* and *Leishmania infantum* were downloaded from the AlphaFold Protein Structure Database [37] (https://alphafold.ebi.ac.uk/download). *Leishmania infantum* is the only *Leishmania* species available in AlphaFold DB. These structures were used to analyze the structural features of RPs.

For *P. falciparum*, gene IDs of RPs analyzed in this study were mapped to UniProt IDs in AlphaFold using information derived from the corresponding CIF files, and only successfully matched RPs were included in subsequent analyses. For *L. infantum*, amino acid sequences were extracted from PDB files using pdb_tofasta function, and clusters were assigned to each sequence following the same procedure used for the outgroup species.

As structural features, we computed the number of residues, the mean pLDDT score, and the mean relative solvent accessibility (RSA) from the PDB files. Secondary structure was assigned using DSSP [38]. DSSP assigns an eight-state classification (SS8). These states were reduced to a three-state classification (SS3) as follows: helix (H, G, I), strand (E, B), and coil (all remaining states), and their counts were normalized by the total number of residues to obtain frequencies.

Furthermore, based on repeat annotations generated by Drepper, .defattr and .cxc files were created and used to visualize the correspondence between repeat regions and protein structures in ChimeraX [39].

## Results

### Overview of repeat architecture analysis

We analyzed amino acid sequences from 12 species of malaria parasites and kinetoplastids, together with two free-living protozoa, a social amoeba, and a yeast species as comparative outgroups (Table 1), quantitatively characterizing repeat architectures using seven repeat-related features derived from existing tools and a newly developed algorithm, as summarized below.

**Length**: amino acid sequence length
**TRcount**: number of TRs
**MaxTRS**: maximum TR region size
**RUP**: purity of the repeat unit sequence
**MaxRUS**: maximum repeat unit size
**Complexity**: complexity of the repeat structure
**TotalRS**: total repetitive region size

### Clustering of RPs

Seven repeat-related features were computed for each RP, and the RPs were clustered based on these features. Figure 3A shows a visualization of the clustering results projected onto the principal component analysis (PCA) space. Hereafter, Clusters 1 through 7 are denoted as C1–C7, respectively.

First, we examined the species composition of each cluster and visualized the results using a heatmap, including 4 comparative species (Fig. 3B). As shown in Fig. 3B, C1 was predominantly composed of *Plasmodium* species among the 12 parasitic species, particularly *P. falciparum* (Pfalciparum3D7). A similar enrichment of C1 was also observed in *D. discoideum*. In contrast, C2 was enriched in *Trypanosoma* species (TbruceiTREU927 andTcruziBrazilA4) and multiple *Leishmania* species (LmajorFriedlin, LdonovaniHU3, LamazonensisPH8, andLmexicanaMHOMGT2001U1103). The degree of enrichment was statistically assessed using a generalized binomial model. In C1, the proportions of Pfalciparum3D7, PvivaxP01, and PknowlesiH were significantly higher than those of other parasitic species ($p < 2\times 10^{-16}$). Similarly, in C2, the enrichment of *Trypanosoma* and *Leishmania* species was highly significant ($p < 2\times 10^{-16}$).

To examine species-specific trends independent of clustering, we visualized the density distribution of RPs for each species in the PCA space (Supplementary Fig. S3). For example, in Pfalciparum3D7, a relatively high density of data points was observed in the regions corresponding to C1, whereas in LmajorFriedlin, a higher density was observed in the regions corresponding to C2.

To characterize the repeat-related features within each cluster, we calculated the mean values of each feature for each cluster from 12 parasitic species and visualized them using a heatmap (Fig. 3C). Both C1 and C2 exhibited large values of TotalRS, indicating that RPs in these clusters generally contained extensive repeat regions. However, these two clusters showed contrasting feature profiles: C1 was characterized by high complexity and low RUP, whereas C2 showed the opposite trend. Therefore, we designate C1 and C2 as the high-complexity, repeat-rich (HCRR) cluster and the low-complexity, repeat-rich (LCRR) cluster, respectively.

C3 and C4 also exhibited relatively large values of TRcount, MaxTRS, and MaxRUS, although to a lesser extent than C1. Among these, C3 showed high RUP and complexity, whereas C4 showed lower values for these features. C5 and C6 were characterized by smaller TotalRS values, with C5 exhibiting higher RUP and C6 exhibiting lower RUP. C7 exhibited uniformly small values for MaxTRS and related features. As discussed below, repeats in C7 were predominantly dispersed rather than TRs, forming a distinct group that was separated from the other clusters in the PCA space.

**Figure 4.**
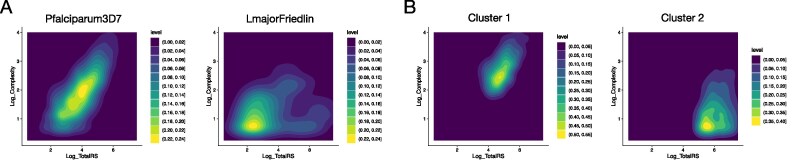
(**A**) Density plots of RPs with logarithm of TotalRS on *x*-axis and logarithm of complexity on *y*-axis, shown for Pfalciparum3D7 (left), and LmajorFriedlin (right). (**B**) Density plots corresponding to panel (A), shown separately for C1 (left) and C2 (right).

To further investigate the relationship between TotalRS and Complexity, we visualized these features for Pfalciparum3D7 and LmajorFriedlin (Fig. 4A). The results for other species are presented in Supplementary Fig. S4. We also visualized the distributions of C1 and C2 (Fig. 4B). The results for other clusters are presented in Supplementary Fig. S5. In Pfalciparum3D7, complexity tended to increase with increasing TotalRS. In contrast, LmajorFriedlin exhibited a substantial number of RPs with a large TotalRS but low complexity. Accordingly, the sequences in C1 and C2 were clearly separated into groups with high and low complexity at high TotalRS values.

**Figure 5.**
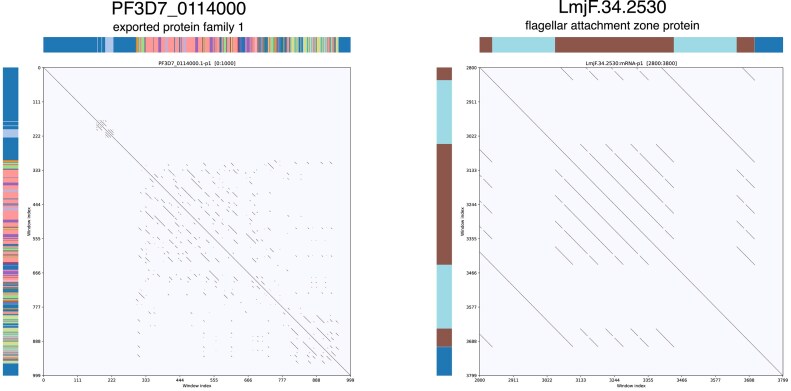
Representative dot plots of RPs in C1 (top) and C2 (bottom). For clarity, only dot plots of a 1000-residue segment from each protein are shown here. Dot plots covering the full-length sequences are provided in Supplementary Fig. S6. The annotations displayed along the top and left margins of the dot plot, together with their colors, represent the clustering results obtained using Drepper.

Representative examples of RPs and their repeat architectures in C1 and C2 are shown as dot plots in Fig. 5 (examples for the other clusters are shown in Supplementary Figs S6 and S7). Numerous off-diagonal elements were observed in both clusters, indicating the presence of extensive repeat regions. In C1, many RPs contained multiple distinct repeats within a single sequence or exhibited highly complex repeat architectures. In contrast, RPs in C2 frequently formed large repeat regions that were close to perfect repeats.

Finally, RPs in C7 generally contained repeats that were spatially dispersed along the sequence, rather than forming contiguous TRs (Supplementary Text). Consequently, their repeat architectures differed substantially from the patterns typically detected using tandem repeat detection tools, and these sequences formed a separate distant cluster in the PCA space.

The proportion of RPs among all proteins in each species is summarized in Supplementary Table S2. *Dictyostelium discoideum* exhibited a relatively high proportion (0.34), and *Plasmodium* species also showed similarly high values, for example, 0.33 in *P. falciparum*, which are larger than that observed in *C. velia* (0.11). In contrast, *Trypanosoma* and *Leishmania* displayed much lower proportions, ranging from 0.014 to 0.036, which are smaller than those observed in *B. saltans* (0.098).

As the overall proportion of repeats increases, the proportion of RPs in the HCRR cluster is expected to increase accordingly. However, RPs in the LCRR cluster were instead enriched in *Trypanosoma* and *Leishmania*, despite their low overall RP proportions. This suggests that the LCRR cluster represents a distinct class of RPs whose enrichment cannot be explained solely by the overall abundance of repeats.

### Signal peptide prediction and analysis

**Figure 6.**
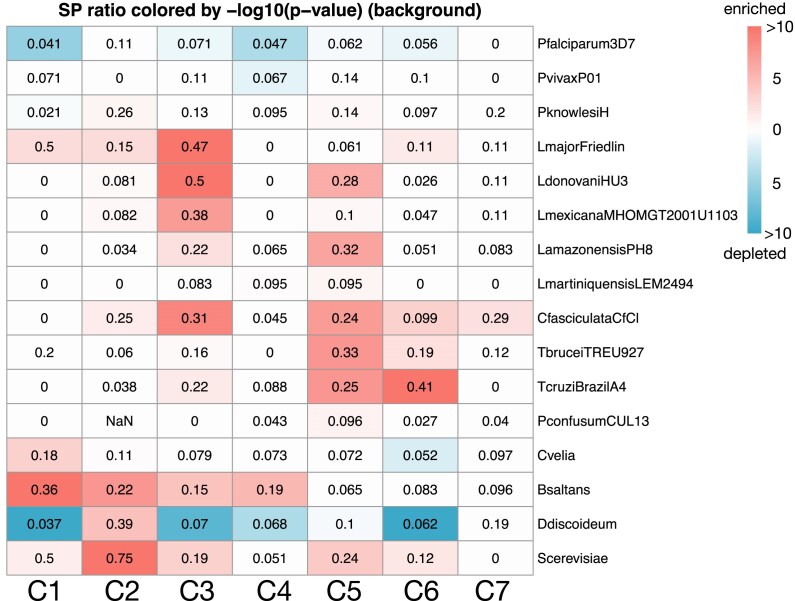
Proportion of RPs predicted to contain signal peptides in each cluster for each species. Colors represent $p$-values from tests of enrichment relative to non-RPs used as a control; red indicates enrichment compared to non-RPs, whereas blue indicates depletion relative to non-RPs.

We examined whether RPs in each cluster, across all species, including outgroups, contain signal peptides. Figure 6 summarizes the proportion of RPs predicted to contain signal peptides in each cluster for each species, along with their enrichment or depletion relative to non-RPs.

In the outgroup species, signal peptides were enriched in certain clusters, such as C2. In contrast, in *Trypanosoma* and *Leishmania*, signal peptides were frequently enriched in C3 and C5. Notably, in *Trypanosoma* and *Leishmania*, the proportion of signal peptide–containing RPs in C2 was consistently lower than that in C3, whereas the opposite trend was observed in the outgroup species.

These results suggest that even for proteins classified as LCRR, the strategies by which they are functionally utilized are species-specific. In particular, *Trypanosoma* and *Leishmania* may have adapted these proteins for distinct biological functions.

### Functional enrichment analyses

We analyzed the functional characteristics of RPs contained in each cluster across 12 parasitic species, based on Gene Ontology (GO) and protein signatures.

**Figure 7.**
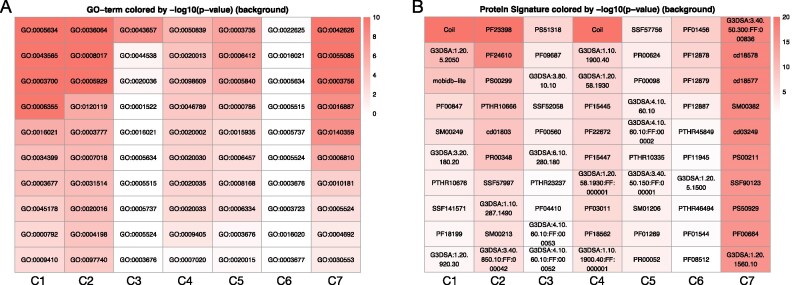
Top 10 GO terms (**A**) and protein signatures (**B**) enriched in each cluster. Colors represent $p$-values. These are the results of enrichment analysis using all other clusters as controls. The results using all genes outside the target cluster, including non-RPs, as controls are shown in the Supplementary Text.

GO enrichment analysis was performed for each cluster using two complementary control sets, and the most significantly enriched terms were visualized (Fig. 7A and Supplementary Fig. S8A). For C1, GO terms, such as nucleus (GO:0005634) and sequence-specific DNA binding (GO:0043565), were highly enriched. In C2, enriched GO terms included ciliary basal body (GO:0036064), microtubule binding (GO:0008017), and cilium (GO:0005929). Other clusters exhibited distinct enrichment patterns: C3 was enriched for host cell (GO:0043657), C4 for cell adhesion molecule binding (GO:0050839), host cell surface receptor binding (GO:0046789), and infected host cell surface knob (GO:0020030), whereas C7 was enriched for ATPase-coupled transmembrane transporter activity (GO:0042626).

We also performed protein signature enrichment analysis for each cluster using two complementary control sets, and the most significantly enriched terms were visualized (Fig. 7B and Supplementary Fig. S8B). In C1, the enriched signatures included the CATH superfamily G3DSA:1.20.5.2050, which contains transcription factors with AP2 domains, as well as the AP2 domain itself (PF00847). In C2, signatures, such as flagellum attachment zone protein 1 (FAZ1; PF23398) and the domain of unknown function DUF7623 (PF24610), were highly enriched. In addition, mucin-like glycoprotein (PF01456) frequently appeared among the most enriched signatures in C5 and C6. When enrichment was assessed using controls that included non-RPs, the protein signature mobidb-lite, which represents intrinsically disordered proteins (IDPs), was consistently enriched across C1 to C6.

C1, which was enriched for *Plasmodium* proteins, showed enrichment of GO terms related to nucleic acid binding and sequence-specific DNA binding; protein signature analysis highlighted domains related to AP2 transcription factors. In *Plasmodium*, the Apicomplexan AP2 (ApiAP2) protein family constitutes the core of sequence-specific transcriptional regulators [40]. Our results suggest that these proteins frequently contain large repeat regions with high complexity.

C2, which was enriched for *Trypanosoma* and *Leishmania*, showed consistent enrichment of flagellum-related functions in both GO and protein signature analyses. This suggests that the proteins associated with these functions tend to contain large repeat regions with low complexity, which may contribute to their biological roles. The DUF7623 domain, which is largely specific to Trypanosomatidae and has been reported to be associated with calpain family cysteine proteases (PF00648), is thought to be involved in parasite life-cycle regulation and parasitism, although its precise function remains unknown.

In C3, host cell appeared to be the most enriched GO term. All genes in this category were derived from *Plasmodium* species, including 11 genes from Pfalciparum3D7, 21 from PvivaxP01, and 18 from PknowlesiH, with a total of 50 genes. Among these, 49 were annotated as *Plasmodium*-exported proteins, many of which were associated with the PHIST (Plasmodium helical interspersed subTelomeric) family or classified as hyp1, hyp2, or hyp11 proteins. The remaining gene encodes an MSP7-like protein (PF3D7_1334300).

In C4, the enrichment of terms, such as host cell surface receptor binding (GO:0046789), was largely attributable to the presence of 24 *P. falciparum* PfEMP1 genes in this cluster. In C5 and C6, enrichment of mucin-like glycoprotein (PF01456), a *Trypanosoma*-derived protein family structurally similar to vertebrate mucins, was observed. These genes encode the core proteins of the parasite mucins and may be involved in interactions with mammalian host cells [41].

Finally, when clusters were compared with controls, including non-RPs, enrichment of the mobidb-lite protein signature was consistently observed. This finding corroborates previous observations that RPs tend to exhibit intrinsically disordered properties.

### Evolutionary analysis

To investigate the evolutionary characteristics of LCRR architectures, we performed a comparative analysis of LCRR proteins across multiple *Leishmania* species. By comparing orthologous repeat regions among *Leishmania* species enriched in C2, we assessed patterns of repeat unit similarity and divergence within low-complexity tandem repeat regions. Twelve orthogroups were identified, in which orthologs from all four *Leishmania* species were assigned to C2. Among these, we focused on three representative orthogroups: Orthogroup 1 containing LmjF.34.2530 (Flagellar attachment zone protein), Orthogroup 2 containing LmjF.16.1660 (Flagellar Member 3), and Orthogroup 3 containing LmjF.33.3070 (Flagellar Member 8).

**Figure 8.**
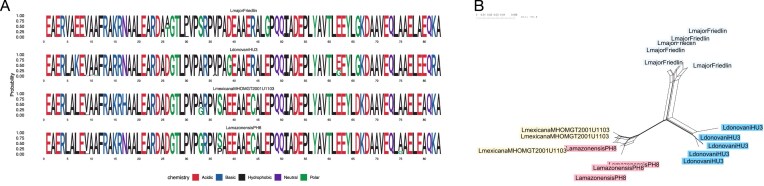
Comparative analysis of sequences in Orthogroup 1 containing LmjF.34.2530 (flagellar attachment zone protein). (**A**) Species-specific sequence logos based on multiple sequence alignment of repeat unit sequences. (**B**) Phylogenetic networks of repeat unit sequences.

First, from the repeat motifs identified in LmajorFriedlin, we selected motifs for which similar repeats were also present in the other three species and enumerated all subsequences within the orthologous proteins that were similar to these motifs. These sequence hits were then subjected to multiple sequence alignments, and sequence logos were generated for each species [Orthogroup 1, shown in Fig. 8 and Supplementary Fig. S9; results for Orthogroup 2 and 3 are provided in the Supplementary Text (Supplementary Figs S10–S12)]. In Orthogroup 1, for example, the first amino acid position was almost uniformly conserved as glutamic acid (E) across all four species, whereas the seventh position differed among the species: E in LmajorFriedlin, K in LdonovaniHU3, and L in both LmexicanaMHOMGT2001U1103 and LamazonensisPH8 (Fig. 8A and Supplementary Fig. S9). These results indicate that repeat unit sequences within orthologs exhibit species-specific differences.

Based on the multiple sequence alignment results, phylogenetic networks of repeat unit sequences were constructed for each orthogroup (Fig. 8B and Supplementary Fig. S12). In all three orthogroups, the repeat unit sequences were clustered predominantly, by species. Moreover, in terms of sequence similarity, LamazonensisPH8 and LmexicanaMHOMGT2001U1103 were relatively closer to each other than to the other two species, reflecting the known species phylogeny.

Analysis of repeat unit sequences revealed that amino acid differences were frequently shared across different repeat units within the same species (Supplementary Fig. S9). Consistently, clustering of repeat unit sequences showed a clear separation by species rather than by repeat unit position or putative ancestral origin (Fig. 8B).

### Structural analysis

**Figure 9.**
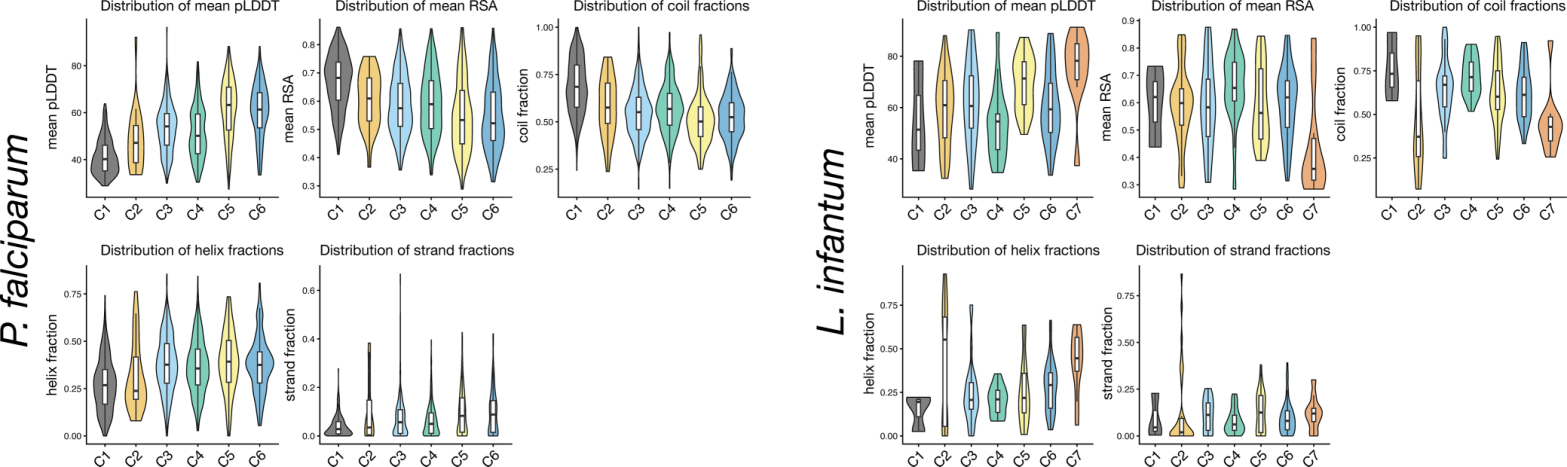
Distribution of structural features across clusters based on AlphaFold predictions. The upper panels show *P. falciparum* and the lower panels show *L. infantum*. For each protein, the mean pLDDT, mean RSA, and the frequencies of coil, helix, and strand were calculated and visualized as distributions.

AlphaFold-based structural features of RPs across clusters in *P. falciparum* and *L. infantum*, the latter being the only *Leishmania* species available in the AlphaFold Protein Structure Database [37], are shown in Fig. 9. The cluster distribution in *L. infantum* is shown in Supplementary Table S3. Consistent with other Leishmania species, the proportion of C2 was relatively high.

In *P. falciparum*, HCRR cluster exhibited a lower mean pLDDT, higher mean RSA, and a higher frequency of coil compared to LCRR cluster (t-test $p$-values $< $ .019, .0057, and .0044, respectively). These characteristics are consistent with typical low-complexity/disordered architectures, indicating that HCRR cluster is enriched for RPs that are unlikely to form stable structures. In contrast, LCRR cluster included RPs with a higher proportion of strand secondary structure, suggesting that some proteins in this cluster adopt more ordered conformations. Among the 27 RPs assigned to LCRR cluster in *P. falciparum*, approximately half of those with structures available in AlphaFold DB were predicted to form repetitive beta-sheet–rich structures.

In *L. infantum*, LCRR cluster showed no statistically significant differences in mean pLDDT or RSA compared to other clusters, indicating comparable overall structural properties. In particular, the proportion of helix was significantly higher in LCRR cluster (*P*$< $ .020), suggesting that RPs in this cluster preferentially form helix-rich structures.

**Figure 10.**
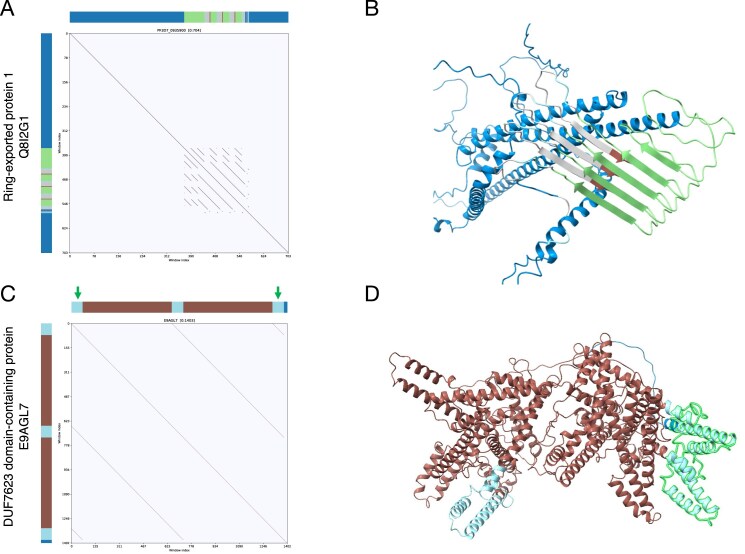
Examples of repeat architectures in RPs belonging to LCRR cluster and their correspondence in three-dimensional structures. Panels (**A**) and (**B**) show *P. falciparum* ring-exported protein 1, whereas panels (**C**) and (**D**) show a *L. infantum* DUF7623 domain-containing protein. For panel (D), the region indicated by the green arrow in panel (C) is highlighted with a green outline in the three-dimensional structure.

We next compared Drepper-based repeat annotations with predicted structures to examine the relationship between repeat sequences and structural features at the individual protein level. For example, in *P. falciparum*, Fig. 10A and B show the dot plot and structural mapping of repeat regions for ring-exported protein 1, a member of LCRR cluster. Drepper analysis revealed a repeat pattern such as AAAABABAB, and corresponding repetitive structural motifs were observed in the predicted structure.

Similarly, Fig. 10C and D illustrate a DUF7623 domain-containing protein from LCRR cluster in *L. infantum*. Drepper identified two complete repeats along with a partial repeat fragment. Notably, among the three occurrences of the short repeat segment, the first and third copies are positioned in close spatial proximity in the predicted structure.

We also investigated the repeat and structural characteristics of *L. infantum* proteins assigned to C3 and predicted to contain signal peptides. As shown in Fig. 3C, proteins in C3 were relatively short and exhibited high RUP and complexity values, suggesting that many of these proteins contain multiple short repeat regions within relatively short protein sequences. Representative dot plots of three examples (surface antigen protein 2, putative surface antigen protein 2, and proteophosphoglycan ppg4) demonstrated that multiple short repeats were frequently observed in these proteins (Supplementary Fig. S13). Comparison with predicted structures revealed that some repeat regions corresponded to repetitive structural elements enriched in beta sheets, whereas others were located in regions lacking confident structural prediction (Supplementary Fig. S13). Notably, in putative surface antigen protein 2, multiple repeats were observed within the repetitive structural regions.

These results demonstrate that integrating Drepper-based repeat annotation with structural models enables detailed investigation of the relationship between repeat sequence patterns and three-dimensional structural organization.

## Discussion

In this study, we designed and quantitatively analyzed the repeat-related features of amino acid sequences from 12 species of *Plasmodium* and some kinetoplastids. Through these analyses, we identified HCRR and LCRR clusters; the HCRR cluster was enriched for *Plasmodium*, whereas the LCRR cluster was enriched for *Trypanosoma* and *Leishmania*.

Signal peptide analysis revealed that LCRR proteins were enriched for signal peptides in the outgroup species, whereas they were less frequently associated with signal peptides in *Trypanosoma* and *Leishmania*. These findings suggest that, in these parasites, proteins in LCRR cluster may be utilized for functions distinct from those in other species.

Functional analyses revealed that genes in HCRR cluster were enriched for AP2 domains, whereas those in LCRR cluster were enriched for flagellum-related GO terms and domains. In malaria parasites, the AP2 family constitutes one of the few classes of transcription factors and plays a critical role in gene expression regulation [40]. Flagellar proteins have long been studied, primarily as motility-related components; however, recent studies have revealed that flagella are involved in a wide range of functions, including migration within host vectors, cell adhesion, and immune evasion [42, 43]. In addition, LCRR cluster was enriched for DUF7623, a domain of unknown function. Therefore, our findings may provide useful insights into the functional roles of these uncharacterized domains.

Furthermore, C3 was enriched for the GO term host cell, with enrichment of genes encoding *Plasmodium*-exported proteins, including those associated with the PHIST family. PHIST proteins may contribute to host cell remodeling during the infection of human erythrocytes and are significantly expanded in *P. falciparum*, although their detailed functions remain poorly understood [44]. In addition, RP proteins containing signal peptides were enriched in C3 in both *Leishmania* and *Trypanosoma*. For example, surface antigen protein 2 (PSA2/GP46) of *L. infantum* was classified into C3; this protein is known to play an important role in host–parasite interactions. In *Leishmania*, the importance of repetitive sequences has been recognized in several major surface and secreted virulence factors, and PSA2 belongs to the superfamily of leucine-rich repeat proteins [45]. Furthermore, PSA2 is also a target of host immune recognition and has been proposed as a vaccine candidate [46]. On the other hand, PSA proteins are highly diversified, with multiple putative surface antigen protein 2 family members being present. The observation that C3 contains proteins involved in host–pathogen interactions in both *Plasmodium* and Trypanosomatidae is particularly intriguing, as it suggests that repeat sequences may have conserved evolutionary and functional significance in processes such as immune evasion and host invasion.

We further investigated why amino acid sequences in LCRR cluster maintain large repeat regions with low complexity by performing evolutionary analyses. One possible explanation for the low complexity is that these repeats were acquired relatively recently and have therefore accumulated only a limited number of mutations. However, comparisons of repeat sequences among orthologs from closely related species revealed species-specific amino acid substitutions, together with marked homogenization of repeat unit sequences within each species. If individual repeat units accumulated mutations independently, analogous to distinct gene sequences, repeat units derived from the same ancestral unit would be expected to show greater similarity across species than repeat units located at different positions within the same species. Under this scenario, repeat units would be expected to cluster according to their ancestral origin. However, our results do not support this expectation. Instead, amino acid differences were shared across different repeat units within the same species, and repeat unit sequences clustered primarily by species rather than by ancestral repeat units (Supplementary Fig. S12). These patterns cannot be explained solely by the independent accumulation of mutations in individual repeat units and instead point to the presence of homogenizing forces acting on repeat unit sequences within each species. It is well established that repetitive sequences can undergo concerted evolution through mechanisms such as unequal crossing-over, leading to coordinated sequence evolution among repeat units [47, 48]. Taken together, our results suggest that concerted evolution contributes to the maintenance of low-complexity repeat architectures in LCRR cluster proteins of *Leishmania*.

TRs composed of identical units (perfect repeats) tend to be less structured. In intracellular parasites, proteins containing perfect repeats are expressed at higher levels during those life-cycle stages that are capable of host cell invasion compared to other stages; these proteins are often associated with functions related to cell invasion [12]. However, it has also been argued that perfect repeats do not necessarily imply specific structural or functional roles, but rather represent signatures of recent evolutionary events [6]. Analysis based on AlphaFold-predicted structures revealed that proteins in the HCRR cluster are generally less likely to adopt stable structures, whereas the LCRR cluster includes many proteins with the potential to form structured conformations. Notably, the types of structures formed differ between *Plasmodium* and *Leishmania*. By visualizing Drepper-derived repeat annotations on predicted structures, we were able to map repeat sequences onto their three-dimensional arrangements, enabling detailed examination of the relationship between repeat architecture and structural organization. This approach is expected to facilitate further insights into the structural and functional significance of repeats, particularly within the LCRR cluster. For example, a substantial number of genes in *Plasmodium* and *Leishmania* remain functionally uncharacterized (Supplementary Table S4), including many repeat-containing proteins. Integrating repeat architecture with structural information may therefore contribute to functional inference for these previously unannotated proteins. The present results alone may be insufficient for determining whether the low complexity of repeat structures is actively maintained by concerted evolution or its functional importance. Future studies are required to elucidate the functional significance of these low-complexity repeat architectures in *Trypanosoma* and *Leishmania*.

Repeat architectures within protein sequences can exhibit highly complex patterns, arising from hierarchical repetition and diversification driven by mutational processes. Existing approaches are often insufficient to fully capture this structural complexity. To address this limitation, we developed a novel algorithm, Drepper, which enables quantitative characterization of repeat-structure complexity (Fig. 2). Although several tools have been proposed to analyze repeats using dot plots or related representations [23, 49–51], Drepper was specifically designed to extract repeat-related features that quantify architectural properties such as repeat-structure complexity. By providing a quantitative and integrative framework for analyzing complex repeat architectures, this approach sheds new light on the diversity and evolution of repeat-containing proteins and offers a foundation for future repeat analyses across diverse biological systems.

In this study, we performed comprehensive repeat analyses using a novel algorithm, Drepper, from multiple perspectives. Drepper is freely available at https://github.com/hmatsu1226/Drepper. Further studies are required to clarify the relationship between repeat architectures and parasitic strategies in protozoa. In this context, the application of advanced computational and bioinformatics approaches will become increasingly important. We anticipate that our study will serve as a foundation for future investigations in this field.

## Supplementary Material

lqag061_Supplemental_File

## Data Availability

Drepper is freely available at https://github.com/hmatsu1226/Drepper and https://doi.org/10.5281/zenodo.20174626.
