# Supplementary material for: Distinct repeat architecture landscapes in the proteomes of protozoan parasites
Source: NAR Genom Bioinform. 2026 Jun 27;8(3):lqag061. doi: 10.1093/nargab/lqag061 (PMC13309793; doi:10.1093/nargab/lqag061)
Supplement: lqag061_Supplemental_File [file lqag061_supplemental_file.pdf]

Supplementary Text for Distinct Repeat Architecture Landscapes in the Proteomes of Protozoan Parasites

May 29, 2026

1 Information of outgroup species

As outgroup species, we included the free-living relative *Bodo saltans*. In addition, the unicellular eukaryotic model organisms *Dictyostelium discoideum* and *Saccharomyces cerevisiae* were included. The sequence datasets used in this study are summarized in Table S1.

Table S1: Information of outgroup species and GenBank assembly

**Abbreviation**	**Species**	**GenBank assembly**
Bsaltans	*Bodo saltans*	GCA_001460835.1
Ddiscoideum	*Dictyostelium discoideum*	GCA_000004695.1
Scerevisiae	*Saccharomyces cerevisiae*	GCA_000146045.2

2 Supplementary information for the evaluation of clustering and Drepper

To evaluate the stability of the clustering, we repeatedly performed k-means clustering independently. Twenty representative results are shown in the Fig. S1. In most cases, the adjusted Rand index compared to the clustering used in the main text was ≥ 0.977, indicating that nearly identical clustering results were obtained. However, in approximately 5% of the cases, slightly different clustering results were observed, as shown in the second column of the fourth row in the Fig. S1. Such cases exhibited larger total within-cluster sum of squares values than most other runs, suggesting that they correspond to local optima. Therefore, the clustering used in the main text can be considered both stable and well-optimized.

To assess the stability of Drepper calculations, we computed Complexity and TotalRS values using simulated sequences. Random amino acid sequences of length 1,000 were generated, and within each sequence, a motif of length 20 was repeated $(e - s)/20$ times in the region from position s to e. Subsequently, m amino acids within this repeat region were randomly substituted. For each condition, 100 such sequences were generated, and Complexity and TotalRS were calculated.

The distribution of Complexity values at $(s, e) = (100, 900)$ for $m = 0, 100$, and 200 are shown in the Fig. S2A. As m increases, Complexity also increases. Moreover, the distributions under each condition are consistent, indicating stable quantification under similar conditions. Next, TotalRS values were computed at $(s, e) = (400, 600)$, $(300, 700)$, $(200, 800)$, and $(100, 900)$ with $m = 25, 50, 75$, and 100, corresponding to approximately 12.5% substitution. The results are shown in the Fig. S2B. In general, TotalRS increases linearly with $(e - s)$, demonstrating that the total repeat region size can be stably quantified under comparable conditions.

3 Supplementary information for clustering of RPs

The density distributions of the 16 species in the PCA space are shown in Fig. S3. In *Plasmodium*, particularly *P. falciparum*, a relatively large number of RPs are located in the regions corresponding to C1. In contrast, in *Trypanosoma* species (TbruceiTREU927 and TcruziBrazilA4) and multiple *Leishmania* species (LmajorFriedlin,

LdonovaniHU3, LamazonensisPH8, and LmexicanaMHOMGT2001U1103), a relatively large number of RPs are distributed in regions corresponding to C2.

The density distributions illustrating the relationship between TotalRS and Complexity across all 16 species and clusters are shown in Figs. S4 and S5, respectively. In *Plasmodium*, the complexity tends to increase with increasing TotalRS. By contrast, in *Trypanosoma* species (TbruceiTREU927 and TcruziBrazilA4) and multiple *Leishmania* species (LmajorFriedlin, LdonovaniHU3, LamazonensisPH8, and LmexicanaMHOMGT2001U1103), a substantial number of RPs exhibit large TotalRS values but low complexity. As described in the main text, C1 and C2 both consist of RPs with large TotalRS values, but differ markedly in complexity, with C1 showing high and C2 showing low complexity. C3 and C4 show intermediate TotalRS values, with C3 exhibiting slightly higher complexity than C4. C5 and C6 generally show low values for both TotalRS and complexity.

Representative examples of RPs from each cluster, visualized using dot plots, are shown in Figs. S6 and S7. RPs in C3 are characterized by moderately large TotalRS values and relatively high complexity. RPs in C5 and C6 generally have short amino acid sequence lengths. In C7, the repetitive sequences are distributed in a dispersed manner rather than forming contiguous tandem repeat regions.

We also calculated the proportion of RPs in each species (Table S2). In general, sequence complexity tends to increase with the total amount of repetitive sequences (TotalRS). Consistent with this trend, species with a high proportion of RPs, such as *Plasmodium* and *D. discoideum*, showed an increased representation of the HCRR cluster

Furthermore, due to the limited availability of *Leishmania* species in the AlphaFold Protein Structure Database, structural analyses were performed using *L. infantum* sequences. The cluster distribution in *L. infantum* is shown in Table S3. Consistent with other *Leishmania* species, the proportion of C2 (corresponding to the LCRR cluster) was relatively high.

Table S2: Proportion of repeat-containing proteins (RP) in each species

Strain	RP proportion
Pfalciparum3D7	0.33
PvivaxP01	0.25
PknowlesiH	0.17
LmajorFriedlin	0.026
LdonovaniHU3	0.021
LmexicanaMHOMGT2001U1103	0.025
LamazonensisPH8	0.019
LmartiniquensisLEM2494	0.014
CfasciculataCfCl	0.053
TbruceiTREU927	0.033
TcruziBrazilA4	0.036
PconfusumCUL13	0.033
Cvelia	0.11
Bsaltans	0.098
Ddiscoideum	0.34
Scerevisiae	0.041

Table S3: Cluster proportions of RPs in *Leishmania infantum*

Cluster	1	2	3	4	5	6	7
Proportion	0.02	0.21	0.15	0.11	0.17	0.28	0.06

4 Supplementary information for functional analysis

The results of the cluster-wise enrichment analysis of GO terms and protein signatures are shown in Fig. S8, where all genes outside the target cluster, including non-RPs, were used as controls.

We also examined the number of genes with unknown function in each cluster and among non-RPs, and summarized the results in Table S4. Here, genes were classified as having unknown function if their gene annotation listed the gene product as “unknown function” or “unspecified product.”

Table S4: Number of proteins with unknown function. For each species and cluster, the number of proteins with unknown function and the total number of proteins are shown, separated by a slash (/).

	C1	C2	C3	C4	C5	C6	C7	non-RP
Pfalciparum3D7	163/467	9/27	118/396	133/464	62/192	67/232	0/1	867/3577
PvivaxP01	60/183	0/3	75/208	130/434	97/296	64/241	0/2	1128/4093
PknowlesiH	11/48	12/19	54/159	74/253	82/203	46/196	0/5	1158/4350
LmajorFriedlin	0/6	4/48	1/30	3/36	3/33	4/55	0/9	619/8207
LdonovaniHU3	0/4	2/37	2/24	2/35	4/32	3/39	2/9	747/8224
LmexicanaMHOMGT2001U1103	0/4	3/49	2/24	1/39	1/29	2/43	1/9	573/7809
LamazonensisPH8	0/2	0/29	0/18	0/31	0/28	0/39	1/12	167/8354
LmartiniquensisLEM2494	0/3	0/5	0/12	0/21	0/42	0/27	0/9	161/8111
CfasciculataCfCl	0/23	1/12	0/45	4/134	2/58	5/192	0/14	227/8577
TbruceiTREU927	0/5	3/83	4/32	0/28	6/57	1/67	0/8	181/8298
TcruziBrazilA4	0/5	0/78	0/40	0/34	1/130	0/111	0/34	105/11527
PconfusumCUL13	2/6	0/0	13/24	15/46	28/52	43/111	13/50	2111/8370

5 Supplementary information for evolutionary analysis

Multiple sequence alignments and species-specific sequence logos for Orthogroups 1, 2, and 3, as discussed in the main text, are shown in Fig. S9, S10, and S11. The phylogenetic networks are shown in Fig. S12. In all orthogroups, multiple positions exhibit species-specific homogenization of amino acid substitutions. For example, in Orthogroup 2, the amino acid at position 11 is homogenized as alanine (A) in LmajorFriedlin and LdonovaniHU3, whereas it is homogenized as threonine (T) in LmexicanaMHOMGT2001U1103 and LamazonensisPH8.

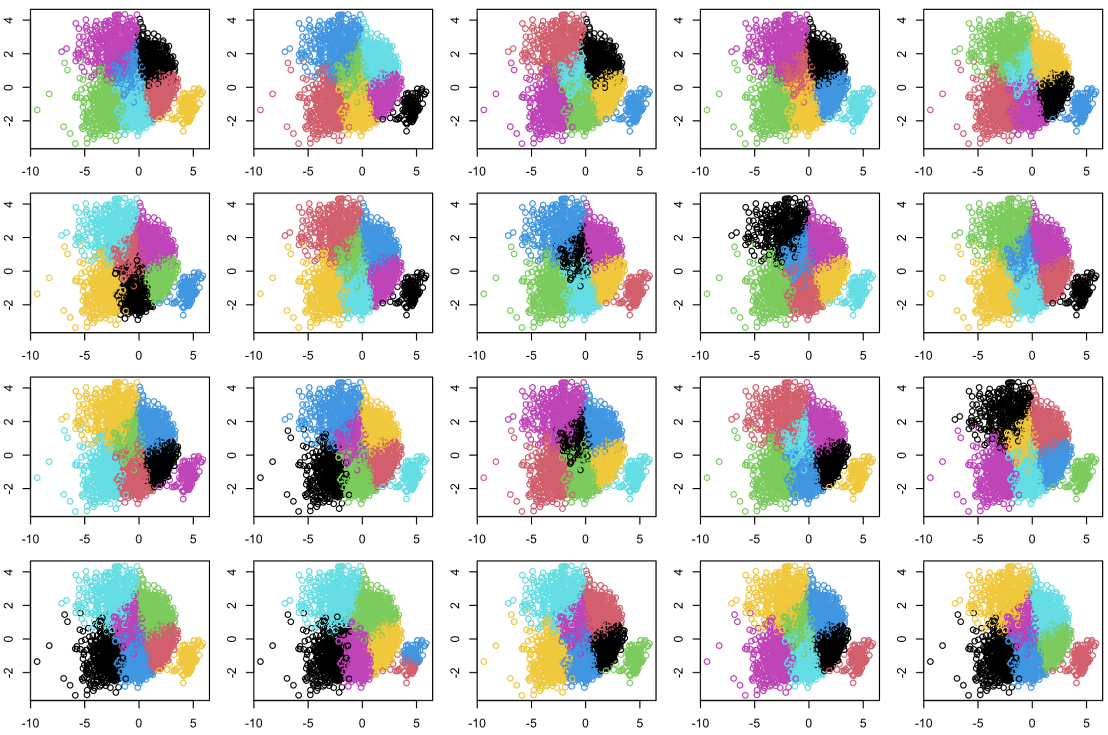

Figure S1: A visualization of clusters obtained by independent k-means clustering projected onto the PCA space.

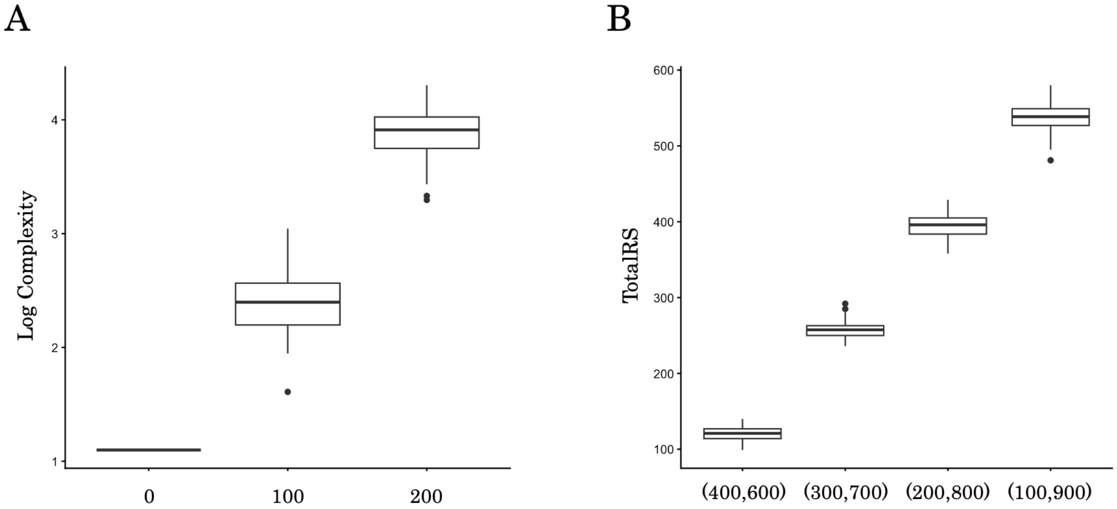

Figure S2: Results of Drepper applied to simulated sequences. (A) Distribution of log(Complexity + 1) at $(s, e) = (100, 900)$ for $m = 0, 100$, and 200. (B) Distribution of TotalRS values at $(s, e) = (400, 600)$, $(300, 700)$, $(200, 800)$, and $(100, 900)$ with 12.5% of amino acids in the repeat region substituted.

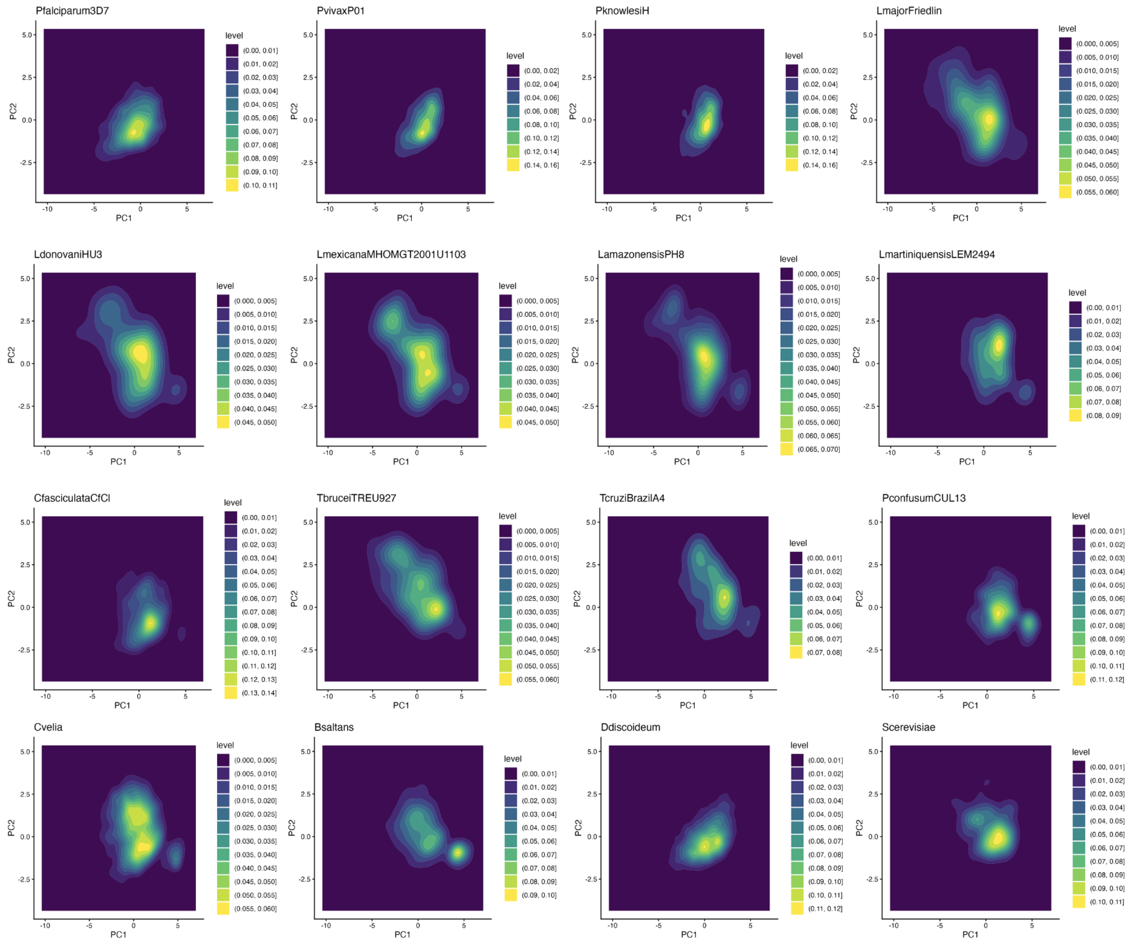

Figure S3: Density distributions of RPs in the PCA space, shown for all species.

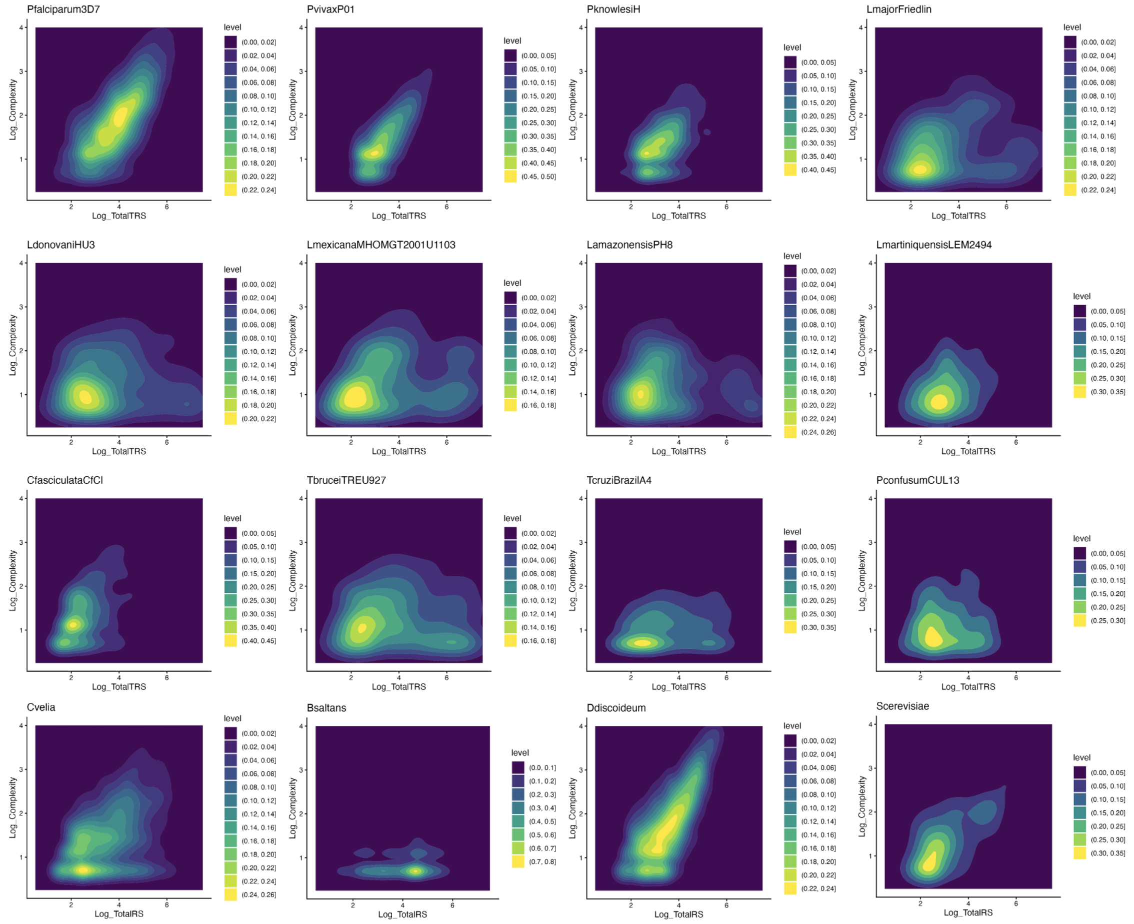

Figure S4: Density plots of RPs for all species, with the logarithm of TotalRS on x-axis and logarithm of Complexity on y-axis.

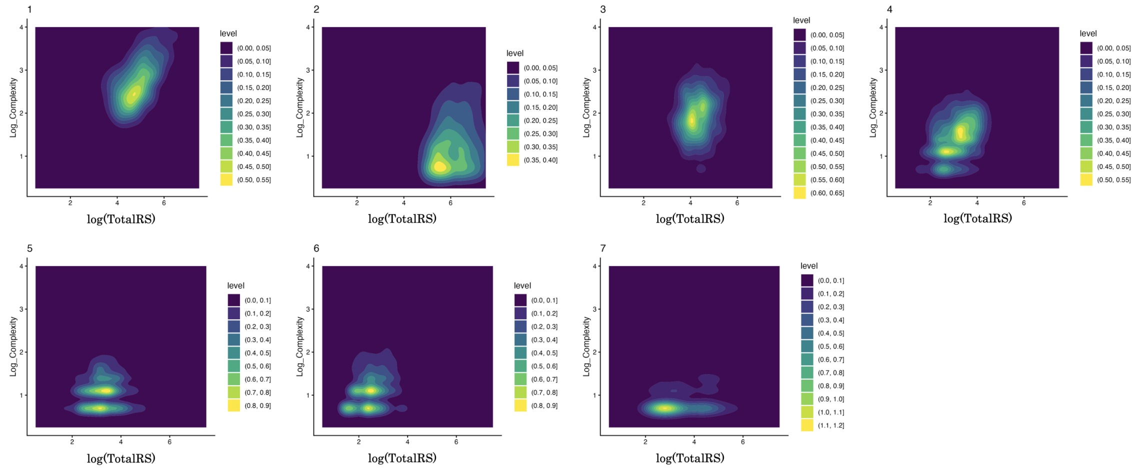

Figure S5: Density plots of RPs for all clusters, with the logarithm of TotalRS on x-axis and logarithm of Complexity on y-axis.

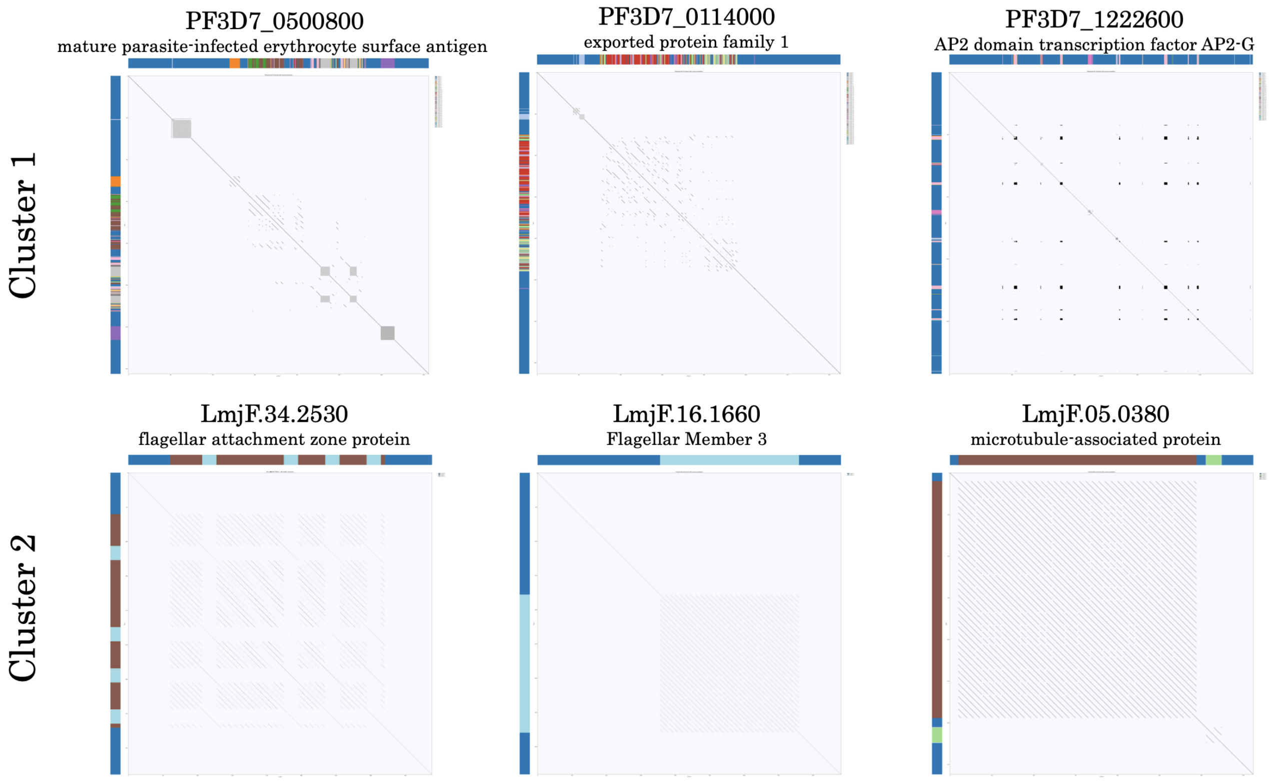

Figure S6: Representative Dot Plots of RPs in C1 (top) and C2 (bottom). The annotations displayed along the top and left margins of the Dot Plot, together with their colors, represent the clustering results obtained by Drepper (see Methods section).

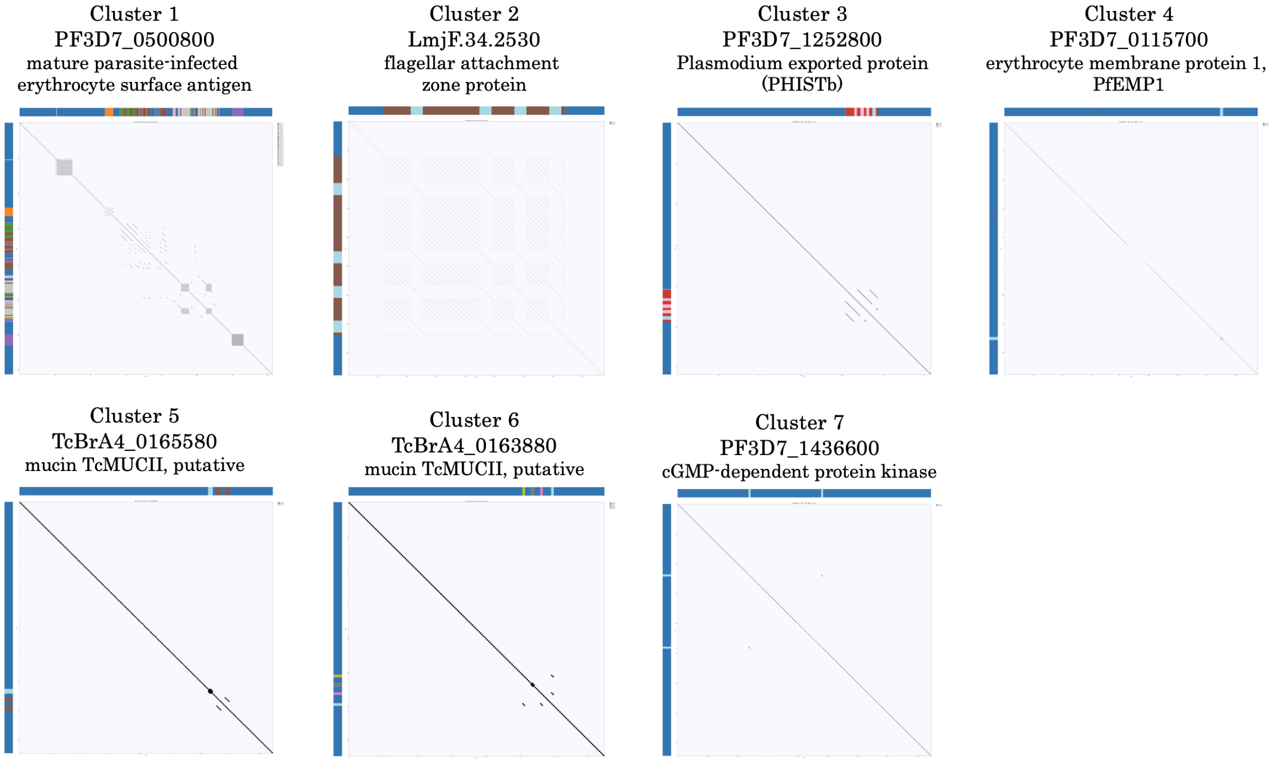

Figure S7: Representative dot plots of RPs in all clusters. The annotations displayed along the top and left margins of the dot plot, together with their colors, represent the clustering results obtained by Drepper (see Methods section).

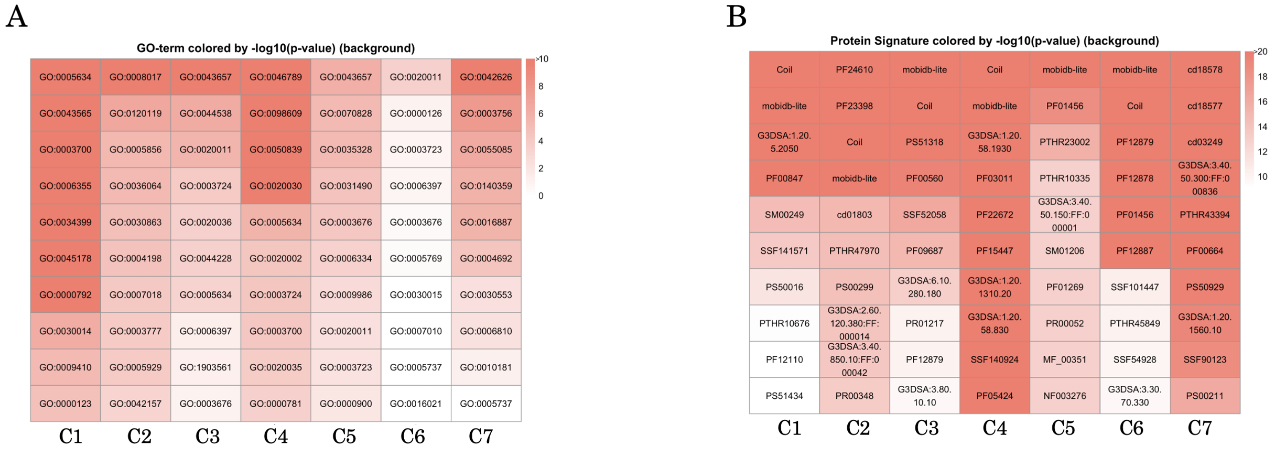

Figure S8: Top 10 Gene Ontology terms (A) and protein signatures (B) enriched in each cluster. Colors represent p-values. These are the results of enrichment analysis using all other clusters as controls. The results using all genes outside the target cluster, including non-RPs, as controls.

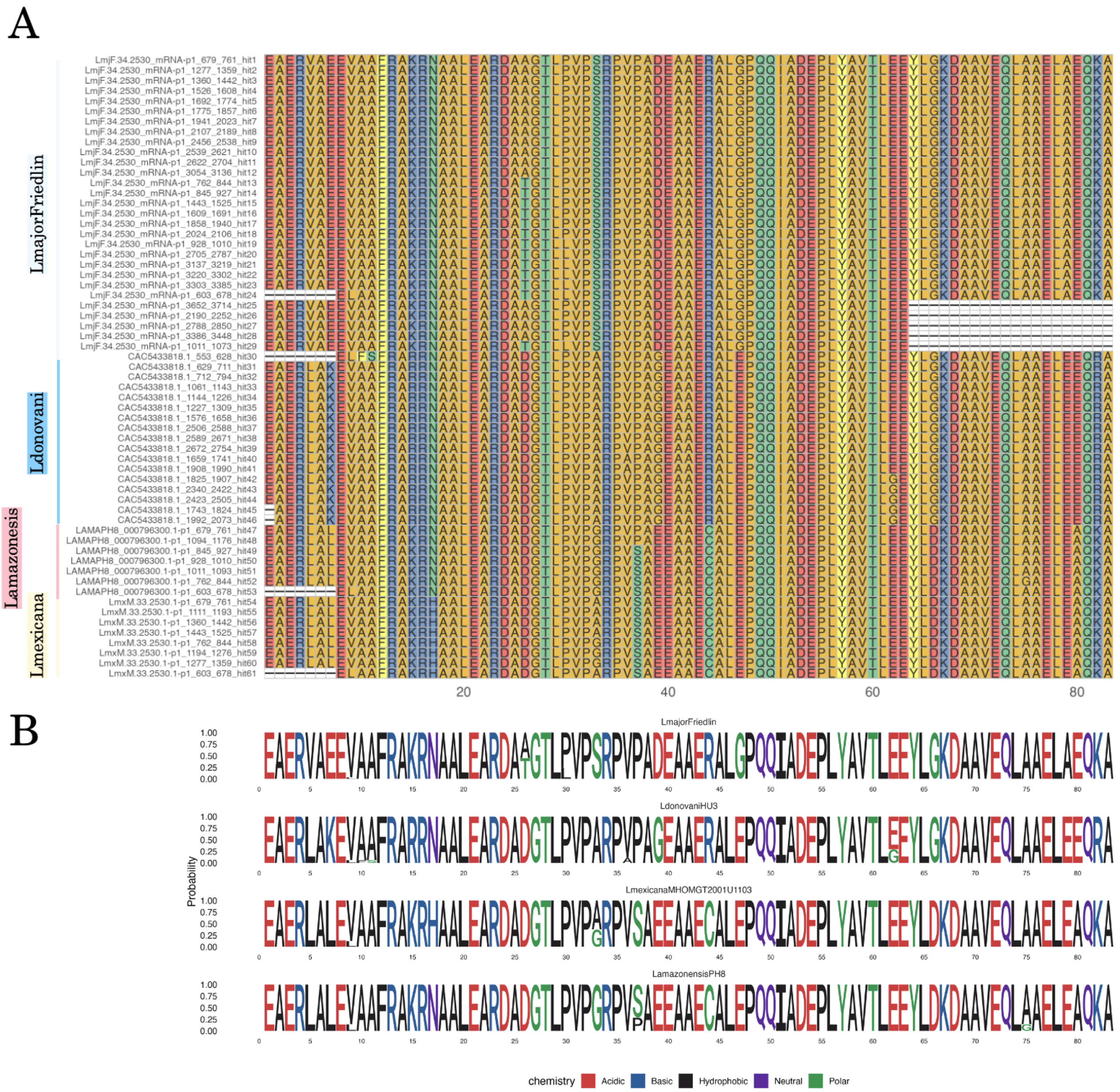

Figure S9: Comparative analysis of sequences in Orthogroup 1 containing LmjF.34.2530 (Flagellar attachment zone protein). (A) Multiple sequence alignment of repeat unit sequences. (B) Species-specific sequence logos.

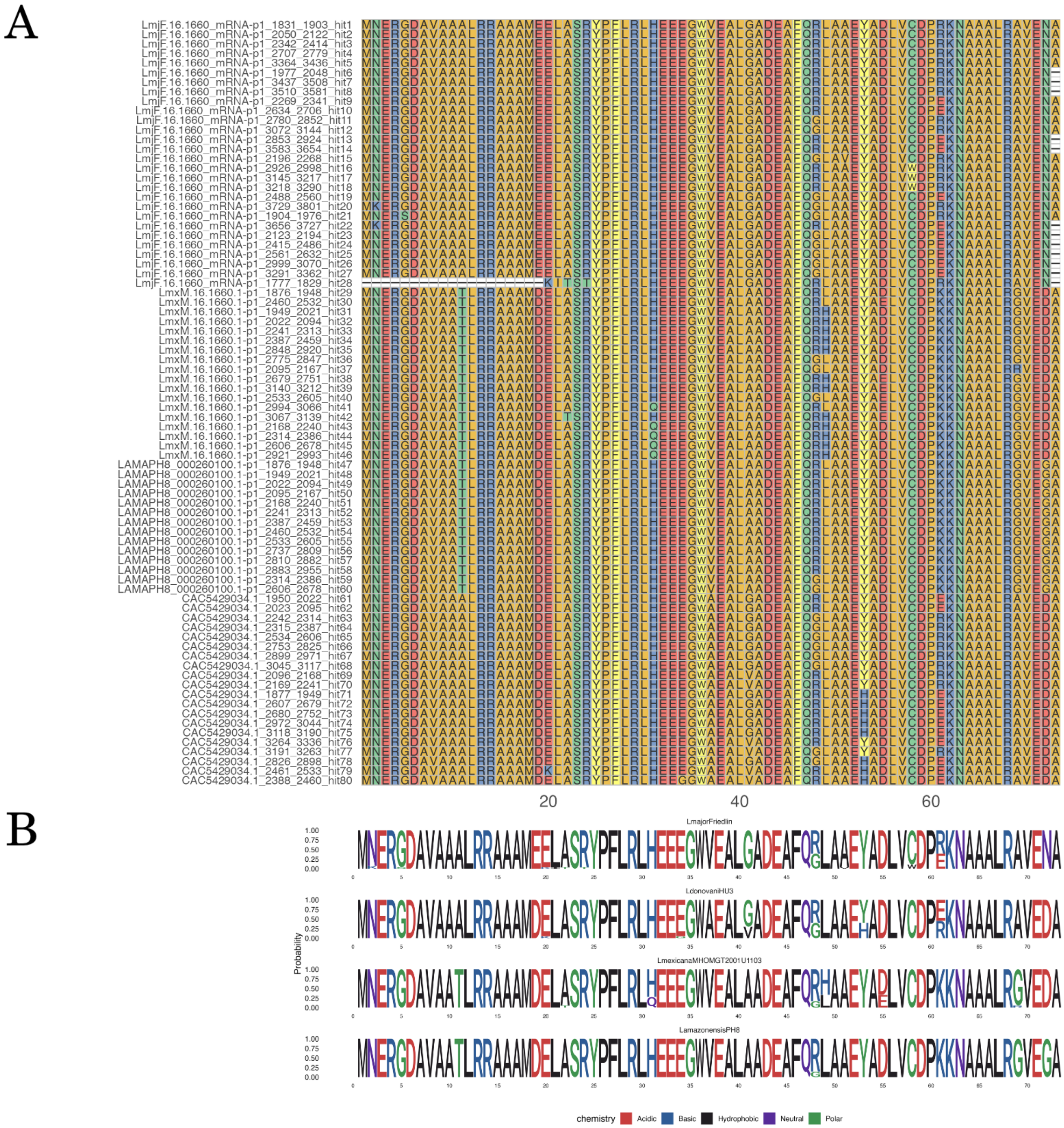

Figure S10: Comparative analysis of sequences in Orthogroup 2 containing LmjF.16.1660 (Flagellar Member 3). (A) Multiple sequence alignment of repeat unit sequences. (B) Species-specific sequence logos.

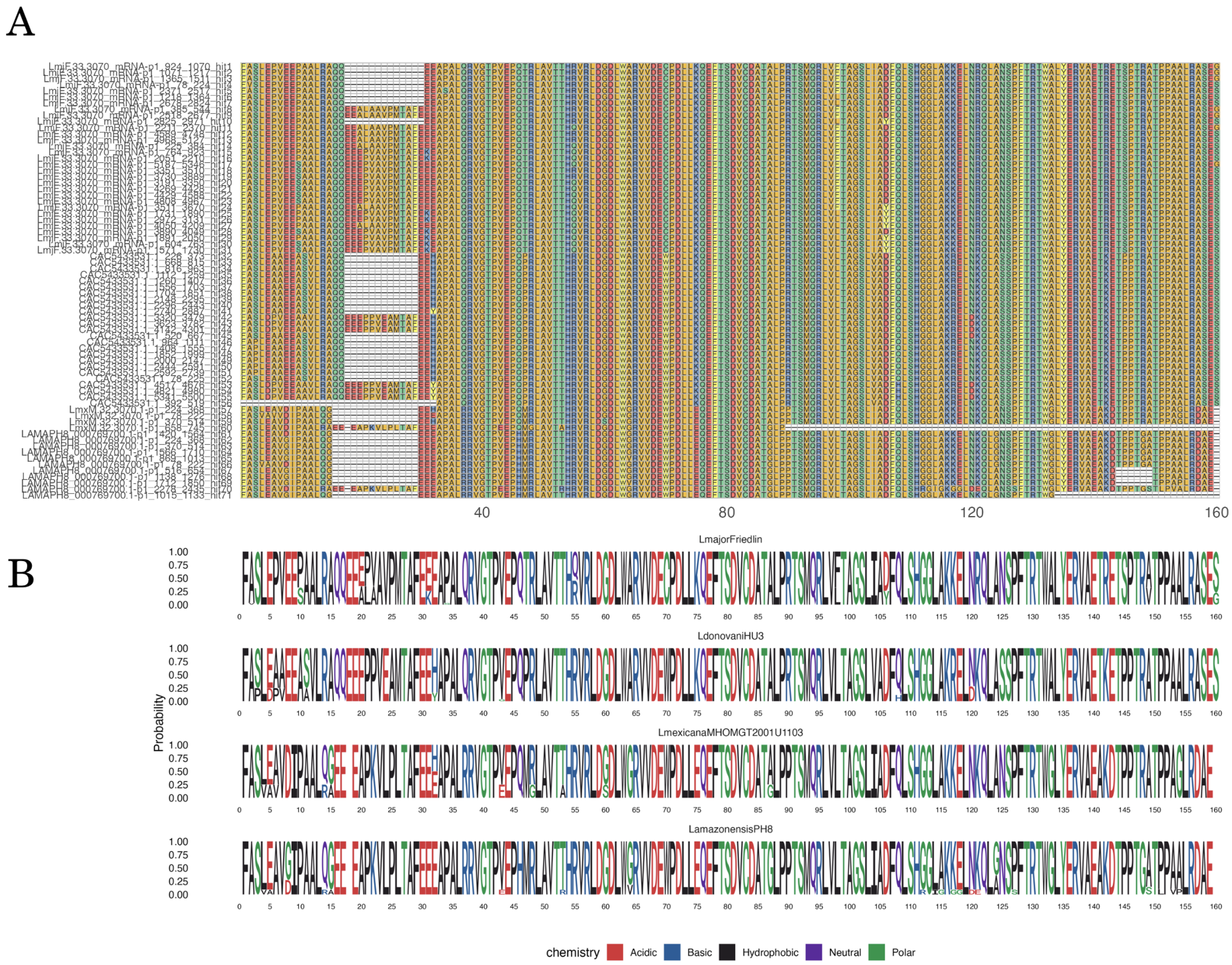

Figure S11: Comparative analysis of sequences in Orthogroup 3 LmjF.33.3070 (Flagellar Member 8). (A) Multiple sequence alignment of repeat unit sequences. (B) Species-specific sequence logos.

A

Orthogroup 1
including LmjF.34.2530,
flagellar attachment zone protein, putative

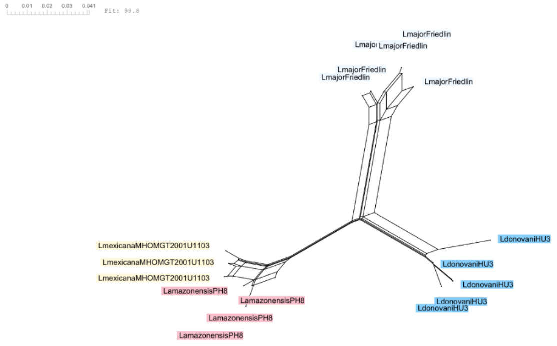

B

Orthogroup 2
including LmjF.16.1660,
Flagellar Member 3

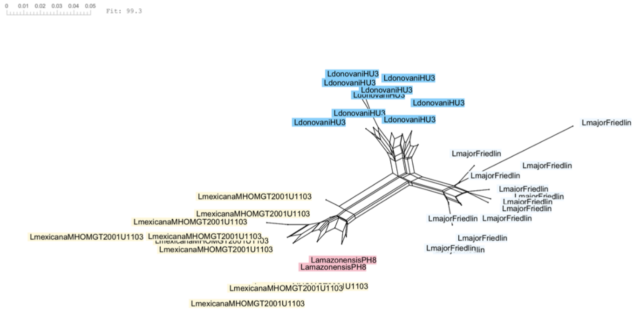

C

Orthogroup 3
including LmjF.33.3070,
Flagellar Member 8

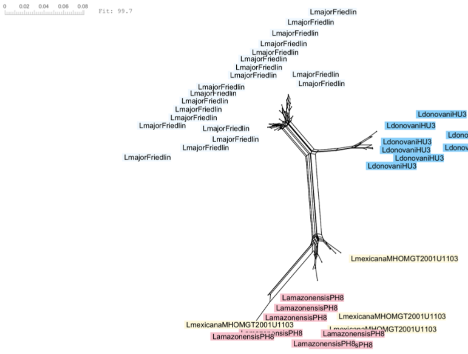

Figure S12: Phylogenetic networks of repeat units in Orthogroup 1 (A), Orthogroup 2 (B), and Orthogroup 3 (C).

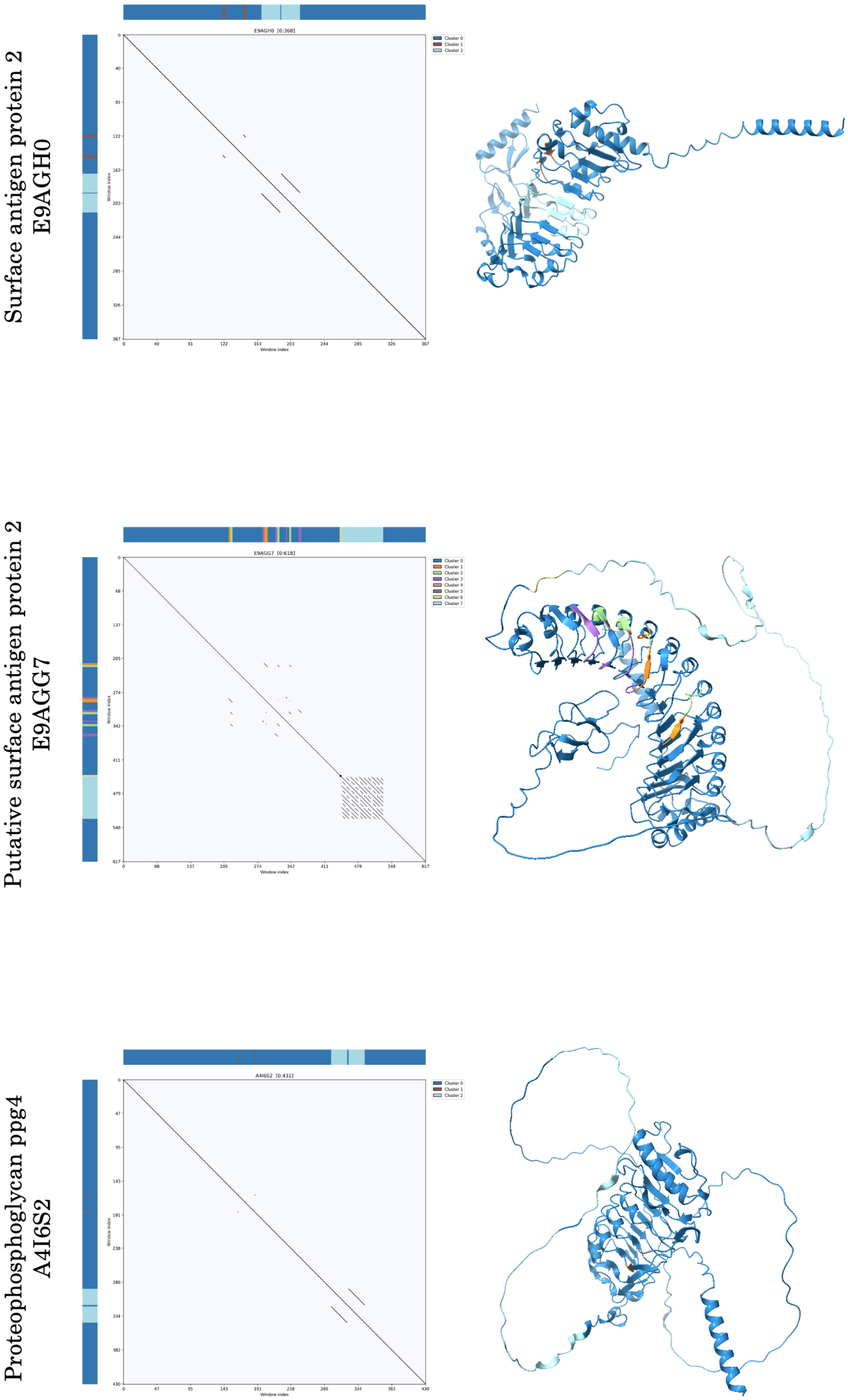

Figure S13: Dot plots (left) and structural representations of repeat regions (right) for *L. infantum* proteins assigned to C3 and predicted to have signal peptides. From top to bottom: surface antigen protein 2, putative surface antigen protein 2, and proteophosphoglycan ppg4.
